# Systematic review of genetic association studies involving histologically confirmed non-alcoholic fatty liver disease

**DOI:** 10.1136/bmjgast-2014-000019

**Published:** 2015-02-17

**Authors:** Kayleigh L Wood, Michael H Miller, John F Dillon

**Affiliations:** 1 School of Medicine, University of Dundee, Dundee, UK; 2 Medical Research Institute, University of Dundee, Ninewells Hospital, Dundee, UK

**Keywords:** NONALCOHOLIC STEATOHEPATITIS, FATTY LIVER, GENETIC POLYMORPHISMS

## Abstract

Non-alcoholic fatty liver disease has an increasing prevalence in Western countries, affecting up to 20% of the population.

**Objective:**

The aim of this project was to systematically review and summarise the genetic association studies that investigate possible genetic influences that confer susceptibility to non-alcoholic fatty liver disease and non-alcoholic steatohepatitis.

**Design:**

The MEDLINE and SCOPUS databases were searched to identify candidate gene studies on histologically diagnosed non-alcoholic fatty liver disease.

**Results:**

A total of 85 articles have been summarised and categorised on the basis of the general pathway each candidate gene is involved in, including lipid metabolism, lipoprotein processing, cholesterol synthesis, glucose homoeostasis, inflammatory response, protection against oxidative stress and whole body metabolism.

**Conclusions:**

The main findings demonstrate a small but consistent association of PNPLA3 with non-alcoholic fatty liver disease and non-alcoholic steatohepatitis. Genetic association studies have investigated general disease susceptibility, histological characteristics, severity and progression. However, further study is required to better elucidate the genetic factors influencing fatty liver disease.

## Introduction

### Overview

Non-alcoholic fatty liver disease (NAFLD) is an increasingly common disorder and a major global burden, affecting up to 30% of the general population and approximately 80% of obese individuals.^1^ NAFLD is frequently associated with the metabolic syndrome including diabetes mellitus, hypercholesterolaemia and obesity, and NAFLD can be divided into simple steatosis (SS), involving lipid accumulation in the liver, and the more severe non-alcoholic steatohepatitis (NASH), accounting for approximately 5% of UK cases.^2^ There are no approved treatments for NAFLD; management is exercise and weight loss.^1^ The mortality in NAFLD is mainly cardiovascular disease; however, NASH causes cirrhosis, end-stage liver disease and hepatocellular carcinoma (HCC).^1^

It has been proposed that the aetiology of NAFLD is multifactorial, involving interacting genetic and environmental factors. Studies have investigated candidate genes for susceptibility to NAFLD and to NASH. This project aimed to produce a systematic review of the candidate gene studies that investigate genetic association with histological characteristics of NAFLD and NASH.

### Diagnosis of NAFLD

NAFLD is defined as “fat accumulation in the liver exceeding 5% of its weight”,^1^ and is a diagnosis of exclusion based on clinical, biochemical and imaging techniques, although the gold standard is liver biopsy.^1^ Diagnosis of NASH requires liver biopsy and is based on histological criteria; there is not a universally accepted system,^1^ but the NAFLD-Activity Score (NAS), by Kleiner *et al*,^3^ has been independently validated and used in research.^1^

## Materials and methods

### Selection criteria and search strategy

Systematic literature searches were performed in accordance with the PRISMA 2009 guidelines^4^ through to 12 May 2014. The MEDLINE and SCOPUS databases were searched for studies evaluating single nucleotide polymorphisms (SNPs) for association with and/or severity of histologically diagnosed NAFLD or NASH, using keywords relating to: (1) disease (MESH “fatty liver”, NASH, NAFLD) and (2) genetic studies (MESH “polymorphism, single nucleotide”, MESH “genetic association studies”, MESH “genetic predisposition to disease”, polymorphism). Results were restricted to human and English language studies. Articles were screened for relevance using the title and abstract. Editorial, correspondence and review papers were excluded. Full-text articles were then obtained for the remaining studies; inclusion required histological diagnosis of NAFLD or NASH, and that fatty liver disease was distinguished from alternate causes of liver disease, including alcoholic, viral, drug-induced and autoimmune aetiologies. Histological diagnosis was used as a standard for study quality, and studies using clinical, imaging or biochemical criteria for NAFLD and NASH were not included. Results were required to be reported using ORs and CIs. A traditional, descriptive review of the literature was performed.

## Search results

### Systematic literature search

**Figure 1:**
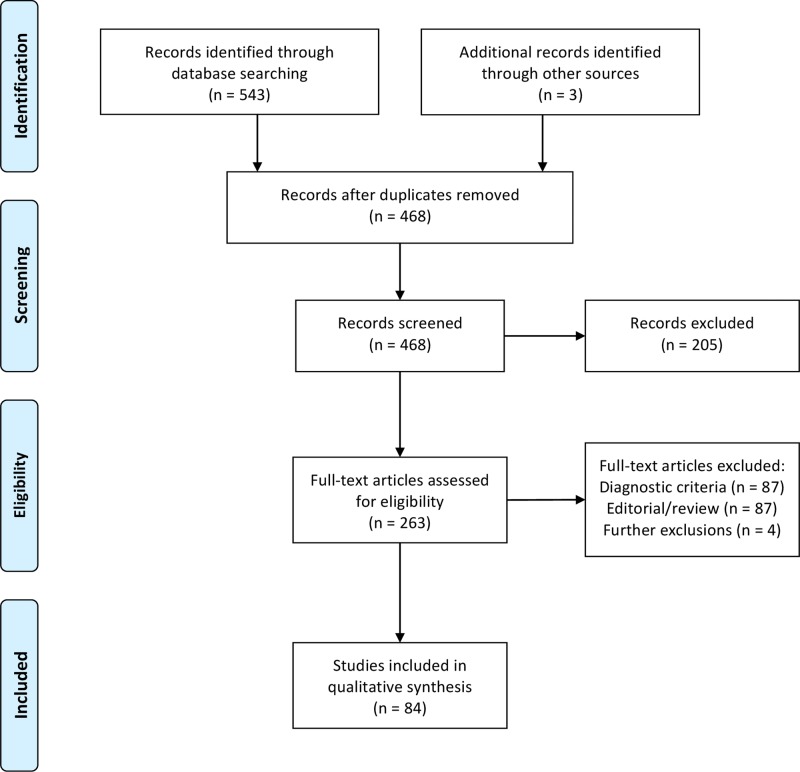
Flow diagram illustrating systematic literature selection process in accordance with PRISMA guidelines.

Literature searches of MEDLINE and SCOPUS databases, through to 12 May 2014, retrieved a total of 465 articles after duplicates were removed. There were 205 articles that were not relevant candidate gene studies, and review, editorial and correspondence articles accounted for 86 papers. Of the genetic studies relating to NAFLD, diagnosis was based on MRI or CT in 25 instances, hepatic ultrasound scan in 48, and 14 papers focused on clinical and biochemical characteristics (figure 1). Four articles were further excluded from the review: in one case, exclusion was due to inconsistency between NAFLD and NASH;^5^ two studies did not report results relating to individual genes;^6^ ^7^ and one study did not exclude fatty liver from the control cases.^8^ One study, published shortly after the search date, was identified by personal communication and was included. Two further studies were manually identified after the search date.

A total of 85 articles investigating over 40 candidate genes were eligible for inclusion in this review. These have been summarised and organised into general pathways, with genome-wide association study (GWAS) papers summarised separately, as shown in table 1. Information relating to study population, number of patient and control participants and candidate genes investigated for each study is summarised in online supplementary table S1. Additional information on candidate genes and polymorphisms, including minor allele frequency, is provided in online supplementary table S2. In all studies, genomic DNA was retrieved from blood samples unless stated otherwise. The results of studies evaluating more than one candidate gene have been described in the relevant sections, where appropriate.

**Table 1:** Summary and pathway overview of candidate genes included in this review

Pathway	Gene symbol^9^	Name^9^	Cytogenetic location^9^	Studies (n)
Lipid metabolism	*PNPLA3*	Patatin-like phospholipase domain-containing 3	22q13.31	11
	*LPIN1*	Lipin 1	2p25.1	1
	*PPARA*	Peroxisome proliferator-activated receptor α	22q13.31	2
	*PPARG*	Peroxisome proliferator-activated receptor γ	3p25.2	5
	*PPARGC1A*	Peroxisome proliferator-activated receptor γ, coactivator 1 α	4p15.2	1
Lipoprotein transport and metabolism	*APOC3*	Apolipoprotein C-III	11q23.3	2
	*APOE*	Apolipoprotein E	19q13.32	4
	*OLR1 (LOX-1)*	Oxidised low-density lipoprotein receptor 1	12p13.2	1
	*PEMT*	Phosphatidylethanolamine *N*-methyltransferase	17p11.2	2
	*MTTP*	Microsomal triglyceride transfer protein	4q23	5
Cholesterol biosynthesis	*SREBPF1*	Sterol regulatory element-binding transcription factor 1	17p11.2	1
	*SREBPF2*	Sterol regulatory element-binding transcription factor 2	22q13.2	1
Oxidative stress	*SOD2*	Superoxide dismutase 2, mitochondrial	6q25.3	2
	*UCP3*	Uncoupling protein 3	11q13.4	1
	*GCLC*	Glutamate-cysteine ligase, catalytic subunit	6p12.1	1
Inflammatory response	*TNF*	Tumour necrosis factor	6p21.33	7
	*IFNL3 (IL28B)*	Interferon λ 3	19q13.2	1
	*IL6*	Interleukin 6	7p15.3	1
	*IL1*	Interleukin 1	2q13	1
	*CD14*	CD14 molecule	5q31.3	1
	*TLR4*	Toll-like receptor 4	9q33.1	1
	*MIF*	Macrophage migration inhibitory factor (glycosylation-inhibiting factor)	22q11.23	1
	*ADRB3*	Adrenoceptor β 3	8p11.23	1
Metabolic hormones	*ADIPOQ*	Adiponectin, C1Q and collagen domain containing	3q27.3	3
	*LEPR*	Leptin receptor	1p31.3	2
Metabolism—transcription factors	*STAT3*	Signal transducer and activator of transcription 3	17q21.2	1
	*CLOCK*	Clock circadian regulator	4q12	1
	*MTHFR*	Methylenetetrahydrofolate reductase (NAD(P)H)	1p36.22	2
	*KLF6*	Kruppel-like factor 6	10p15.1	1
	*CHUK*	conserved helix-loop-helix ubiquitous kinase	10q24.31	1
Hepatic iron accumulation	*HFE*	Haemochromatosis	6p22.2	6
	*TMPRSS6*	Transmembrane protease, serine 6	22q12.3	1
Profibrogenic factors	*AGT*	Angiotensinogen	1q42.2	1
	*AGTR1*	Angiotensin II receptor, type 1	3q24	2
	*TGFB1*	Transforming growth factor, β 1	19q13.2	1
	*SERPINE 1 (PAI-1)*	Serine peptidase inhibitor, clade E (nexin, plasminogen activator inhibitor type 1), member 1	7q22.1	1
Glucose homoeostasis	*IRS1*	Insulin receptor substrate 1	2q36.3	1
	*ENPP1 (PC-1)*	Ectonucleotide pyrophosphatase/phosphodiesterase 1	6q23.2	2
	*GCKR*	Glucokinase regulator	2p23.3	1
	*TCF7L2*	Transcription factor 7-like 2	10q25.2-q25.3	1
Neurological	*CNR1*	Cannabinoid receptor 1	6q15	1
	*CNR2*	Cannabinoid receptor 2	1p36.11	1
	*NCAN*	Neurocan	19p13.11	1
Miscellaneous	*TM6SF2*	Transmembrane 6, superfamily member 2	19p13.3-p12	3
	*SPINK1*	Serine peptidase inhibitor, Kazal type 1	5q32	1
	*ABCB11*	ATP-binding cassette, subfamily B (MDR/TAP), member 11	2q31.1	1
	*NR1H4*	Nuclear receptor subfamily 1, group H, member 4	12q23.1	1
	*CYP2E1*	Cytochrome P450, family 2, subfamily E, polypeptide 1	10q26.3	1
	*NR1I2*	Nuclear receptor subfamily 1, group I, member 2	3q12–q13.3	1
	*FABP2*	Fatty acid-binding protein 2, intestinal	4q26	1
	*SLC27A5*	Fatty acid transport protein 5	19q13.43	
	*CHDH*	Choline dehydrogenase	3p21.1	1
	*SAMM50*	Sorting and assembly machinery component	22q13.31	1
GWAS	*FDFT1*	Farnesyl diphosphate farnesyltransferase 1	8p23.1	1
	*EFCAB4B*	EF-hand calcium-binding domain 4B	12p13.32	1
	*LTBP3*	Latent transforming growth factor β (TGF-β) binding protein	11q13.1	1
	*SLC2A1*	Solute carrier family 2, member 1	1p34.2	1
	*COL13A1*	Collagen, type XIII, α 1	10q22.1	1
	*LYPLAL1*	Lysophospholipase-like 1	8q11.23	1
	*PPP1R3B*	Protein phosphatase 1, regulatory subunit 3B	8p23.1	1
	*PARVB*	Parvin, β	22q13.31	1
	*GCKR*	Glucokinase regulator	2p23.3	2

Highlighted genes indicate associations that have been replicated in more than one histologically characterised cohort.

GWAS, genome-wide association study.

## Candidate gene studies

### Lipid metabolism

#### PNPLA3

The patatin-like phospholipase domain-containing protein 3 gene (*PNPLA3*), also known as adiponutrin, encodes a lipase enzyme expressed in adipocytes.^9^ The gene was first identified as a candidate for NAFLD susceptibility in a GWAS by Romeo *et al*,^10^ which found a significant association with the rs738409 polymorphism (not included in this review as fatty liver was assessed using 1H-MRI). The rs738409 variant is thought to eliminate the lipase activity of the enzyme.^11^

**Figure 2:**
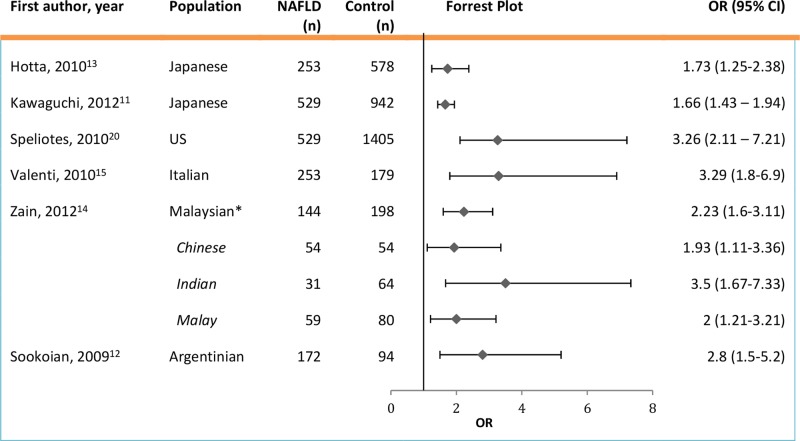
Studies evaluating the association between non-alcoholic fatty liver disease (NAFLD) and rs738409 of *PNPLA3*. *Combined Malaysian population including Chinese, Indian and Malay ethnic subgroups.

A number of candidate gene studies have replicated the association of *PNPLA3* polymorphisms in different populations (figure 2). The effects of rs738409 on histological characteristics, disease severity and NASH diagnosis have also been investigated.

Sookoian *et al*^12^ performed a case–control study evaluating rs738409 polymorphism on an Argentinian population of 172 patients with NAFLD, compared with 94 controls. Discrete trait analysis showed the G allele to be associated with NAFLD compared with controls, and this relationship persisted with multivariate logistic regression, independently of age, sex, body mass index (BMI) and insulin status (HOMA-I). Of the patients with NAFLD, 103 were biopsied, 40 had SS and 63 had NASH. The G allele variant was found to be significantly associated with disease severity (GG>CG>CC) and with NASH compared with SS, with reported OR 1.88 per G allele (95% CI 1.03 to 3.43). Increased steatosis was observed with the homozygous variant (GG) compared with the heterozygous variant, which was subsequently more severe than observed for the wild type genotype.

Hotta *et al*^13^ evaluated the effects of rs738409 on a Japanese cohort of 253 patients with NAFLD and 578 healthy population controls. Of the patients with NAFLD, 189 were diagnosed with NASH and 64 with SS. The study demonstrated a significant dose-dependent relationship of SNP frequency between the NAFLD and control groups, and between patients with NASH and SS. Histologically, the variant allele had an additive effect with increased fibrosis stage, but no association was found with steatosis.

Zain *et al*^14^ evaluated the rs738409 polymorphism in a mixed ethnicity Malaysian group of 144 patients with NAFLD and 198 healthy population controls, with subgroups of Malay, Chinese and Indian ancestry. Results indicated that NAFLD was significantly associated with the risk G allele with an overall OR 2.34 which remained significant after age and gender were controlled for, and the association was different between the ethnic subgroups as demonstrated in figure 2. The NAFLD group was histologically subdivided into patients with SS, NASH with fibrosis score <2, and NASH with fibrosis score of 2+. When NASH was compared with SS, the variant G allele was found to be associated with NASH (OR 2.64). Histological characteristics of steatosis, lobular inflammation, and hepatocellular ballooning and fibrosis grade were further analysed; the GG genotype was significantly associated with fibrosis stage but no other features. Patients with at least one G allele were found to be more likely to have a fibrosis score of ≥2 (OR 1.95), suggesting a relationship with disease severity.

Valenti *et al*^15^ investigated the association of the *PNPLA3* I148M variant with histological NAFLD in a European population, comprised of 321 UK and 253 Italian patients. The variant G allele was found to be significantly more prevalent in the Italian patients with NAFLD compared with 179 healthy, population-matched controls, and participants with homozygous variant genotype had an increased risk of NAFLD compared with wild type. In the combined population, the variant G allele was found to be an independent predictor for grade 2–3 steatosis, and had a dose-dependent effect on fibrosis stage >1, independently of steatosis. The variant allele and GC/GG genotypes were also associated with the presence of NASH in the combined and UK cohorts, independently of BMI, steatosis and diabetes. The study also investigated allele transmission in 71 Italian family trios, observing that the variant allele was preferentially transmitted compared with the wild type.

In the same year, Valenti *et al*^16^ studied the rs738409 polymorphism with histological severity of NAFLD in an Italian paediatric cohort (n=149). The study found that severity of steatosis was associated with the variant GG genotype (OR 18.86), independently of age, body mass and metabolic syndrome. The homozygous variant was also associated with lobular inflammation, ballooning and perivenular fibrosis, but not periportal fibrosis.

Verrijken *et al*^17^ investigated PNPLA3 rs378409 on a cohort of 470 overweight and obese Caucasian patients, of whom 287 underwent liver biopsy. Histological characteristics were evaluated in the biopsied subgroup, with heterozygotes and variant homozygotes found to have a risk of increased steatosis, lobular inflammation, severity of inflammation and ballooning, with sex-adjusted and age-adjusted OR of 7.41 (CI 3.235 to 16.697), 1.86 (CI 1.046 to 3.321), 3.5 (CI 1.712 to 7.150) and 2.78 (CI 1.514 to 5.119), respectively. Fibrosis was not found to be associated.

Guichelaar *et al*^18^ also evaluated the association of PNPLA3 rs378409 with histological severity on obese patients. A cohort of 144 predominantly female obese patients underwent biopsy; 12 had normal histology, 60 had SS and 72 were found to have NASH. The rs378409 G allele was found to be associated with NASH when compared with the combined normal and patients with SS, with multivariate OR 2.5 (CI 1.1 to 5.3).

Rotman *et al*^19^ evaluated six polymorphisms including rs738409 in 894 adult and 223 paediatric patients within the US NASH CRN cohort. The adult cohort was compared with a population control group of 336 Caucasian men aged 50 years and over. Results showed three variants: rs738409 and rs2281135 in *PNPLA3* and rs2143571 in nearby *SAMM50* were significantly associated with NASH compared with controls. The remaining three variants investigated: rs11597390 (*CPN1)*, rs11597086 and rs11591741 (*CHUK)* were not significantly associated with NAFLD. These were associated with increased fibrosis, although whether this specifically relates to fibrosis in NASH requires further investigation.

Disease severity was further investigated, with univariate analysis showing a significant association between rs738409 allele frequency and features of steatosis and both portal and lobular inflammation. Variant allele frequency was not significantly associated with histological fibrosis or cellular injury but was associated with more severe disease in a dominant pattern. This relationship persisted after factors of age, sex, BMI, diabetes and alcohol intake were controlled for. Patients with the GG and GC genotypes were more likely to have a moderate-to-severe steatosis score (≥2; OR 1.46), Mallory-Denk bodies (OR 1.55), lobular inflammation (OR 1.84) and portal inflammation (additive OR 1.57). Multivariate analysis showed a highly significant association of the G allele with fibrosis in an additive manner, which remained after adjustment for histological parameters of steatosis, inflammation and ballooning, suggesting an independent variable. Fibrotic bridging was associated with an adjusted OR of 1.50 for each G allele. The variant allele was also associated with an increased NAS in a dominant pattern on univariate and multivariate analyses. Polymorphisms rs2281135 and rs2143571 on chromosome 22 had similar associations with NAS. Furthermore, 438 adult patients with definite histological NASH were compared with 82 with SS and the control cohort (n=336), but the rs738409 was not significantly different between SS and NASH groups. In contrast to results found by Valenti *et al*,^16^ the rs738409 SNP was not associated with any histological parameters in paediatric patients.

Speliotes *et al*^20^ studied 12 polymorphisms at seven loci in patients in the US NASH CRN cohort. A total of 592 patients of European ancestry were compared with a control group comprised of 1405 US and European patients enrolled in the Myocardial Infarctions Genetics Consortium (MIGen) study. The MIGen study used a control group to limit the influence of cardiovascular disease status. The rs738409 was found to be significantly associated with both NAFLD and overall histological components (steatosis >5%, lobular inflammation, hepatocyte ballooning, NASH diagnosis and fibrosis) compared with control. However, the histological features are highly inter-related within the NASH CRN cohort, so the case–control associations may not be reflective. *PNPLA3* rs2294918 and rs2281135 SNPs were also assessed, but no association was observed after controlling for the I148M variant. One limitation to this study is that the MIGen control group was not assessed for the presence of liver disease. Case-only analysis was performed within the NASH CRN cohort, and showed that rs738409 was associated with increased lobular inflammation ≥2, but not hepatocyte ballooning or NASH diagnosis. When steatosis distribution was evaluated, patients with the variant allele were less likely to have perivenular centred steatosis. The authors hypothesise that this may be related to diffuse fat deposition and progression to advanced disease.

Petta *et al*^21^ evaluated the influence of *PNPLA3* rs738409 polymorphism on NAFLD severity in a Sicilian population. Of 160 patients with NAFLD, 99 had NASH, 15 did not have NASH and 46 were described as indeterminate. The study found the *PNPLA3* rs738409 variant was associated with moderate-severe steatosis (grade 2–3) by univariate analysis, but the relationship did not persist after Bonferroni correction. Multivariate logistic regression showed the homozygous variant rs738409 genotype was significantly associated with a higher score when using a recessive model.

Corbin *et al*^22^ investigated 21 candidate genes on 446 patients in the Duke University Health Systems NAFLD Clinical Database and Biorepository. The study found that *PNPLA3* was significantly associated with increased histological steatosis only, with an additive effect. The study did not observe any associations with *ADIPOQ*, *CHDH, MTHFD1, PEMT, PPARG* and *STAT3* genes; further information regarding these genes is given in relevant sections.

Gorden *et al*^23^ studied a cohort of 1092 US patients who underwent liver biopsy during bariatric surgery. Of the 1092 patients histologically assessed, 748 had evidence of NAFLD (187 of these diagnosed with NASH), with no evidence of steatosis in the remaining 344 patients who were used as the control group. A number of candidate genes including *PNPLA3* were investigated, and rs738409 was found to be significantly associated with histological features of increased steatosis and hepatocyte ballooning. The *LYPLAL*, *GCKR* and *PPP* variants also studied were not associated with an increased risk of lobular inflammation, ballooning or fibrosis, but a trend towards increased steatosis was observed.

Sookoian *et al*^24^ performed a systematic review and meta-analysis of the *PNPLA3* rs738409, comparing wild type (CC) and variant (GG) genotypes. Six of the studies with histological NAFLD assessment described above were included: Valenti,^15^ Valenti,^16^ Sookoian *et al*,^12^ Speliotes *et al*,^20^ Rotman *et al*,^19^ Hotta *et al*.^13^ Meta-analysis showed that NASH was more frequently associated with the variant genotype compared with the wild type. The variant genotype was also associated with increased inflammatory scores when four of these studies were considered, and significantly associated with fibrosis on the basis of five studies. The study also commented that the heterozygous risk was similar to the homozygous variant and suggested a dominant model for the risk of severe histological features.

#### LPIN1

Lipin is involved in triglyceride synthesis, lipid metabolism and adipocyte differentiation.^9^ The rs13412852 variant was investigated by Valenti *et al*^25^ in two Italian cohorts of 142 paediatric and 115 adults with NAFLD, compared with 337 healthy controls. With regard to the paediatric patients, the variant was significantly less frequent in NAFLD compared with control. Histologically, the homozygous variant genotype was associated with reduced fibrosis (OR 0.29, CI 0.11 to 0.66), and trended towards reduced NASH prevalence. No significant differences were noted between the adult patients and control group, although trends towards fewer NASH and reduced fibrosis were observed with the homozygous variant. The results suggest a protective role for the minor allele; however, the paediatric results should be interpreted cautiously as were compared with an adult control group.

##### PPAR

The peroxisome proliferator-activated receptors are a group of nuclear receptors involved in regulating the transcription of genes involved in metabolic pathways.^26^ Stimulation of *PPARG*, along with coactivator *PPARAGCA1*, upregulates the expression of genes involved in adipocyte differentiation and fatty acid storage, whereas *PPARA*, expressed in the liver, stimulates fatty acid catabolism.^26^

Gawrieh *et al*^27^ investigated the *PPARG* Pro12Ala and C1431T variants in a US population. DNA was extracted from liver biopsy samples of 212 patients with NAFLD, and blood samples from 62 controls. Results did not reach statistical significance for allele frequency or histological characteristics. Genetic modelling and haplotype analysis of the two *PPARG* variants showed that patients with both minor alleles (GT) had a lower risk of NAFLD, but also increased inflammation and advanced fibrotic change, when compared with patients with both major alleles (CC). However, these results should be interpreted with caution, as there was a low successful genotyping rate of 65% from the biopsy samples.

Rey *et al*^28^ studied the effect of the *PPARG* Pro12Ala polymorphism in German patients with fatty liver disease, including 100 patients with alcoholic liver disease and 263 patients with NAFLD. Genomic DNA from fatty liver patients was extracted from biopsy samples and compared with 259 blood samples of healthy controls. A slightly increased but non-significant relationship was seen in variant allele incidence between NAFLD and control groups, and there was no association with histological inflammation in NAFLD.

Dongiovanni *et al*, 201028a investigated the *PPARG* (Pro12Ala) and *PPARA* (Leu162Val) variants in a biopsy-proven NAFLD cohort of 202 Italian patients, compared with 346 healthy controls. Analysis showed that the allele frequency was not significantly different between NAFLD and control, and no significant difference was observed in histological characteristics.

The same variants of the *PPARA* and *PPARG* genes (Leu162Val and Pro12Ala, respectively) were also investigated by Domenici *et al*.^29^ A cohort of 103 Brazilian patients with NAFLD was compared with 103 healthy controls; of the NAFLD group, 89 had histological NASH and 14 had SS. There was no significant difference in allelic frequencies between NAFLD and control, but the Ala *PPARG* variant was less common in the NASH group compared with the control group. Histologically, the wild type *PPARA* genotype (LeuLeu) was found to be associated with more severe fibrosis.

Yoneda *et al*^30^ performed a case–control study investigating 15 SNPs in the *PPARGC1A* candidate gene, in a Japanese cohort of 115 patients with NAFLD and 441 healthy population controls. Results showed that the T allele of rs2290602 was significantly associated with NAFLD (OR 2.73) in the dominant mode when compared with control. Within the NAFLD group, 65 patients had histological NASH, and 50 had SS; a trend towards increased T allele frequency in the NASH group was noted, however this did not reach statistical significance, possibly a result of the small sample size.

Sahebkar^31^ performed a meta-analysis of the PPARG Pro12Ala polymorphism. Seven studies were included in the quantitative review, including the four histologically proven studies described above. Results of the meta-analysis showed no protective or predisposing effect of the variant in any genetic model. When the results of Gawrieh *et al*^27^ were excluded as an outlier due to high BMI, the variant was significantly associated with NAFLD, and the author proposes the effects of obesity may complicate the relationship.

### Lipoprotein transport and processing

#### APOC3, APOE and ORL1

The apolipoprotein C-III, encoded by *APOC3* gene on chromosome 11, is a very low-density lipoprotein (VLDL) component that functions to inhibit lipoprotein lipase and hepatic lipase.^9^ Mutations in *APOC3* are known to be associated with hypertriglyceridaemia and it is hypothesised that this protein delays catabolism of triglyceride-rich particles and is involved in hepatic lipid processing.^9^ The *APOE* gene on chromosome 19 encodes apolipoprotein E, the main apoprotein of the chylomicron that is involved in endocytosis and clearance of chylomicron and VLDL particles by the liver.^9^ Mutations in this protein are known to be associated with familial dysbetalipoproteinaemia.^9^ Oxidised low-density lipoprotein (LDL) receptor 1 is encoded by *ORL1* on chromosome 12.^9^ The receptor binds oxidised LDL, which is then internalised and degraded in the liver.^9^

Valenti *et al*^32^ investigated *APOC3* polymorphisms T-455C and C-482T in a cohort of 758 European (Italian and UK) patients with NAFLD. The case–control study between the Italian patients with NAFLD and 316 healthy Italian controls found no protective effect of the *APOC3* wild type, and no significant association with histological characteristics or NASH diagnosis was observed. Verrijken *et al*^17^ also investigated the rs2854117 variant of APOC3, finding no significant associations with disease severity, fibrosis or other histological characteristics.

Sazci *et al*^33^ evaluated apolipoprotein E polymorphisms in 57 Turkish patients with NASH, compared with 245 healthy controls. The study found that the *APOE*ε3 isoform was associated with an increased risk of NASH, whereas the heterozygous *APOEε3ε4* was protective.

De Feo *et al*^34^ investigated the three major isoforms of *APOE* on an Italian population of 310 patients with NAFLD (109 biopsy proven) and 422 population controls. The case–control study found that at least one *APOEε4* allele significantly reduced the risk of NAFLD (OR 0.51, CI 0.28 to 0.93) when compared with the homozygous wild type. Histological assessment in the biopsied subgroup found no significant association with *APOE* genotype and fibrosis.

Gambino *et al*^35^ compared 29 Italian patients with NASH with 27 controls but found no significant difference in *APOE* polymorphisms; however, this study has less power due to small sample size. Musso *et al*^36^ also investigated *APOE* isoforms on a larger Italian population, comparing 78 patients with NAFLD, 34 of whom were biopsied and diagnosed with NASH, to 156 healthy controls. *APOE* genotypes were not found to be significantly different. The study also compared 40 Italian patients with NASH with 40 *ORL1* genotype-matched controls and concluded that the *ORL1* IVS4–14 G allele was associated with the severity of steatosis, necroinflammation and fibrosis.

#### PEMT

Phosphatidylethanolamine *N*-methyltransferase (PEMT) is an enzyme in the liver that catalyses the conversion of phosphatidylethanolamine to phosphatidylcholine,^9^ a substance that is required for VLDL secretion. Dong *et al*^37^ evaluated the prevalence of the *PEMT* V175M allele in a Japanese population between 107 patients with NASH, a normal control population (n=150) and a cohort of 100 patients with chronic hepatitis C. Both the M allele frequency and M/− genotype were significantly increased in the NASH participants when compared with the control and patients with chronic hepatitis C. There were no significant differences in genotype or allele frequency between the normal and hepatitis C controls, indicating that the association observed is likely to be more specifically related to NASH than viral hepatitis.

#### MTTP

Microsomal triglyceride transfer protein (MTTP) is a heterodimeric protein involved in triglyceride, phospholipid and cholesterol ester transport,^9^ and lipoprotein assembly. The *MTP* gene located at 4q23 encodes the 88kD large subunit of MTTP, and mutations in this protein are associated with familial abetalipoproteinaemia.^9^ The encoded variant of *MTP* −493, with C to G transversion, undergoes less transcription than the wild type protein.^38^

Namikawa *et al*^38^ studied the variant −493 G allele of *MTP* in 63 biopsy proven patients with NASH with 150 healthy controls in a Japanese population. The MTP allele and homozygous variant genotype were both found to be significantly associated with NASH compared with controls. The variant genotype was also associated with increased steatosis and increased histological grade of NASH when compared with the heterozygous carriers.

Gambino *et al*^35^ described above also investigated the frequency of the homozygous *MTP* variant between patients with NASH and controls. No significant difference in allele frequency was observed between Italian patients with NASH and controls, but within the NASH group, the severity of histological steatosis, inflammation and fibrosis was increased in patients with the variant genotype when compared with heterozygotes and homozygous wild type patients. However, these results should be interpreted with caution due to the limited sample size of 27 patients.

Carulli *et al*^39^ performed a candidate gene study in an Italian population investigating the three candidate genes *MTP*, *PCIKI* and *IL6*. The study compared 114 patients with NAFLD, including 59 who underwent biopsy, against 79 healthy population controls. With regard to *MTP*, the case–control study was not significant, and there was no association with histological characteristics or NASH diagnosis within the biopsied group. The study also found no association with the *PCIK* I121Q variant, and results pertaining to *IL6* are described in the relevant section.

Oliveira *et al*^40^ evaluated the *MTP* −493 (C/G) polymorphism in a cohort of 139 Brazilian patients with NAFLD, comprised of 45 with SS and 86 with NASH. The *MTP* G allele was not found to be associated with NAFLD when compared to 141 controls or with NASH when compared to SS.

El-Koofy *et al*^41^ investigated the association of *MTTP* and *SOD2* SNPs with NAFLD in a paediatric Egyptian cohort. A total of 76 children with NAFLD were compared with 20 age-matched and sex-matched healthy controls. Within the NAFLD group, 33 underwent liver biopsy, and of these, 8 had SS, NASH was present in 7 cases, and the remaining 18 samples showed normal histology. The homozygous −493 *MTP* genotype (TT) was not present in any of the 15 patients with NASH or SS. No association was reported with the *SOD2* 118T/C variant, but when the homozygous wild types of *MTP* (GG) and *SOD* (TT) were combined, the study reported a significant risk of NASH compared with controls (OR 54, CI 4.1 to 707). These results should also be treated with caution as only seven patients diagnosed with NASH were studied.

### Cholesterol biosynthesis

#### SREBP

Sterol regulatory element-binding proteins are leucine zipper transcription factors that regulate the transcription of enzymes involved in cholesterol biosynthesis.^9^ Musso *et al*^42^ investigated the effect of the rs11868035 variant of *SREBP* factor 1C on histological severity in 42 biopsy-proven Italian patients with NAFLD. The wild type and heterozygous genotypes were found to be associated with an increased severity of steatosis, necroinflammation, increased NAS, increased fibrosis score and increased prevalence of NASH when compared with the mutant genotype. Multivariate analysis showed this *SREBP1C* polymorphism to be independently associated with both severe steatosis and NASH. In a separate study, the same group evaluated the effect of polymorphisms in *SREBP* factor 2 in biopsied patients with NAFLD.^43^ The study reported that patients with heterozygous and homozygous variant genotypes demonstrated an increased severity of histological characteristics and NASH diagnosis compared with wild type patients. The *SREBPF2* variant allele was found to be an independent predictor of NASH within these patients (OR 2.92, CI 2.08 to 4.18).

### Oxidative stress

There are a number of theories regarding the role of cellular oxidative stress as a factor contributing to the development of NAFLD and NASH. It is therefore unsurprising that a number of studies have investigated genetic variants that might reduce the ability of hepatocytes to protect against oxidative stress, including polymorphisms in the *SOD2*, *UCP3* and *GCLC* genes.

#### SOD2

The superoxide dismutase 2 (*SOD2*) gene on chromosome 6 encodes the enzyme manganese superoxide dismutase.^9^ This is located on the outer mitochondrial membrane, and catalyses the conversion of superoxide by-products of oxidative phosphorylation to hydrogen peroxide and oxygen.^9^ Mutations in *SOD2* have been previously associated with idiopathic cardiomyopathy, certain cancers and motor neuron disease.^9^ The rs4880 C47T polymorphism of *SOD2* is a C to T transversion that results in a valine to alanine substitution in the mitochondrial-targeting region of the protein and may alter the tertiary protein structure.^44^

Al-Serri *et al*^44^ conducted a linkage study on 71 Italian families and found that the C47T SNP was preferentially transmitted. The study then evaluated the C47T *SOD2* polymorphism in a European cohort of 510 patients with NAFLD. The study showed the *SOD2T* variant was associated with advanced fibrosis (scores of >1) with an additive effect. When the *PNPLA3* rs738409 was controlled for, the *SOD2* was still associated with the degree of histological steatosis, and was an independent risk factor for advanced fibrosis. However, the *SOD2* allele did not affect the diagnosis of NASH.

Namikawa *et al*^38^ performed a case–control study between 63 patients with NASH and 150 healthy controls in a Japanese population to evaluate the *SOD2* C47T allele. The study found the homozygous variant genotype (TT) was significantly associated with NASH, however the T allele frequency was not. The *MTP* polymorphism was also investigated (described above), and the proportion of patients with NASH with both GG (*MTP*) and TT (*SOD2*) genotypes was significantly increased compared with controls.

#### UCP3

Uncoupling protein 3 encoded by the *UCP3* gene is a mitochondrial proton carrier located at 11q13,^9^ primarily expressed in skeletal muscle.^9^ The protein is a mitochondrial anion carrier and facilitates the transport of anions from the inner to outer mitochondrial membrane, thus reducing the mitochondrial membrane potential.^9^ The protein is considered to be protective against oxidative stress from β oxidation of fatty acids.^9^ Aller *et al*^45^ investigated the −55C/T polymorphism of *UCP3* in a cohort of 39 obese patients who underwent liver biopsy. The heterozygous genotype was found to be associated with an increase in both moderate-to-severe histological inflammation and moderate-to-severe steatosis.

#### GCLC

The catalytic subunit of glutamate-cysteine ligase (GCLC) is involved in the synthesis of glutathione (GSH).^9^ GSH is a tripeptide that counteracts cellular oxidative stress from reactive metabolites through thiol group reduction.^46^ Oliveira *et al*^40^ evaluated the −129C/T *GCLC* polymorphism in a cohort of 139 Brazilian patients with NAFLD, 45 with SS and 86 with NASH. The investigators observed a significant association between the T allele of *GCLC* with patients with NASH when compared with the SS group.

### Inflammatory response

A key histological component that differentiates SS from more severe disease is the inflammatory response. Polymorphisms of genes encoding components of the immune response, including proinflammatory cytokines such as tumour necrosis factor (TNF), the interleukin (IL) family of molecules and macrophage migration inhibitory factor (MIF), have been investigated for potential associations with NAFLD and disease progression. Toll-like receptor 4 (TLR4), a pathogen recognition molecule, and the CD14 molecule expressed by macrophages are lipopolysaccharide (LPS) cell surface antigens involved in the immune response.^9^

#### TNF

Valenti *et al*^47^ investigated two TNF polymorphisms (*TNFA* 238 and *TNF2* 308) in 99 patients with NAFLD (53 biopsied) and compared with 172 healthy controls. The allele frequency and genotype of *TNF2* was not different between NAFLD and controls; however, both the *TNFA* variant allele and genotype were significantly increased in NAFLD. In the biopsied subgroup, 35 patients had NASH whereas 18 had SS. A trend towards a higher prevalence of *TNF* polymorphisms in NASH subgroup was observed when compared with those with SS, but this did not reach statistical significance, which may be due to the small sample size. Yang *et al*^48^ investigated these two TNF variants in obese children; histologically, 61 were normal and used as control, 17 had SS and 33 had NASH. No significant difference was observed.

Hu *et al*^49^ also investigated the same variants, comparing a cohort of 189 Chinese patients with NAFLD, 44 diagnosed histologically, to 138 healthy controls. Neither variant was homozygous in either the patient or control groups. The heterozygous genotype and A allele frequency of the *TNFA* variant was found to be significantly associated with NAFLD compared with control, with relative risk 2.19. No histological associations were observed in the biopsied subgroup, although the sample size for comparison was small.

Chowdhury *et al*^50^ evaluated the effect of the −308 and −238 TNF variants on fibrosis in NAFLD, investigating 29 Indian patients with biopsy-proven NAFLD. The variants were not found to be associated with fibrosis severity; however, DNA amplification only worked in 25 cases, reducing the sample size.

Aller *et al*^51^ investigated the −308TNF polymorphism on histological characteristics in 66 patients with NAFLD and 213 obese controls. Variant frequency was not significantly different between patients and controls, although within the patient group those carrying the heterozygous variant (n=15) had significantly more severe inflammation and fibrosis than those with wild type genotype; however, the sample size is small.

Tokushige *et al*^52^ investigated six *TNF* polymorphisms in a Japanese population of 102 patients with NAFLD and 100 controls, and found no significant differences between the NAFLD and control groups. Of the patients with NAFLD, 36 had SS and 66 had NASH: the −1031C and −863A variants were significantly more frequent in the NASH group compared with SS, but there were no associations with histological features.

Wong *et al*^53^ investigated the role of three variants of TNF in a Chinese population. A cohort of 79 patients with NAFLD, comprising 61 individuals with SS and 18 patients diagnosed with NASH, was compared with 40 healthy population controls. The results showed no significant differences in the frequency of the three *TNF* polymorphisms investigated.

##### Interleukins

The study by Carulli *et al*^39^ described above also evaluated the *IL-6*-174 G/C SNP, comparing 114 patients with NAFLD against 79 controls. Of the NAFLD group, 59 patients underwent liver biopsy; 29 had NASH, 30 had NAFLD. The case–control study showed that *IL-6* GC and CC genotypes were significantly more prevalent in the NAFLD group, and furthermore, multivariate logistic regression showed the association of GC and CC genotype with NAFLD was independent of age, gender and BMI (OR 4.116, CI 1.126 to 15.048). Histological features were compared between biopsied patients with NASH and NAFLD. The *IL-6* CC and CG variants were associated with NASH (OR 7.035, CI 1.167 to 42.394), but not histological characteristics.

Petta *et al*^21^ investigated the rs12979860 and rs8099917 variants of *IFNL3*, also known as *IL28B*, on histological features in 160 Sicilian patients with NAFLD. The study found the wild type (CC) genotype of rs12979860 was independently linked to moderate-severe lobular inflammation, remaining significant after Bonferroni correction. The rs12979860 wild type was also associated with severe fibrosis by univariate analysis, but not after Bonferroni correction, and multivariate logistic regression was not significant. The rs8099917 was not reported to show any significance.

Nozaki *et al*^54^ investigated the association between −511T/C variant of *IL1B* and B3 adrenergic receptor 190T/A polymorphism on a cohort of 63 Japanese patients with NASH. Case–control study showed that the *IL-1T* allele and TT genotype were significantly more frequent in the NASH group compared with 100 healthy controls. The *B3ADRC* mutant allele was also found to have a higher prevalence in the NASH group.

#### MIF

Akyildiz *et al*^55^ investigated the association of the −173 G/C MIF variant with NAFLD and severity. Ninety-one patients with NAFLD stratified as 37 with NASH, 44 probable NASH and 10 SS were compared with 104 healthy controls. MIF variant genotype and allele were not significantly different between NAFLD cases and control, or within the NAFLD groups.

##### LPS receptors

In a brief communication, Brun *et al*^56^ describe the effect of polymorphisms in genes encoding the TLR4 and CD14 LPS receptors on 28 patients with NAFLD, including 21 with NASH, who were compared with 52 healthy controls. No significant association was seen with *TLR4*; however, the *CD14* homozygous mutant genotype was not present in the seven patients with NAFLD without NASH, and was significantly increased in patients with NASH than controls.

### Metabolism

#### Hormones

Adiponectin, encoded by *ADIPOQ*, and leptin are hormones secreted by adipocytes. Adiponectin is involved in the regulation of energy homoeostasis and metabolism, particularly through enhancement of insulin sensitivity.^9^ Reduced plasma levels of adiponectin have been associated with features of the metabolic syndrome including NAFLD. Leptin acts through its receptor, encoded by *LEPR*, to stimulate satiety and reduce food intake, and also increase energy expenditure.^9^ Studies have investigated polymorphisms in *ADIPOQ* and *LEPR* for association with NAFLD.

##### ADIPOQ

Wong *et al*^53^ studied four SNPs in the adiponectin gene, comparing a cohort of 79 Chinese patients with NAFLD with 40 population controls. Within the NAFLD group, 61 patients had SS and 18 were diagnosed with NASH. The results showed no significant difference in any of the four *ADIPOQ* polymorphisms studied between the NAFLD and control groups, or histological severity within the NAFLD group.

Tokushige *et al*^57^ investigated adiponectin polymorphisms +45 and +276 in a Japanese population. A cohort of 119 patients with NAFLD was compared with 115 healthy population controls. There were no significant differences in the frequency of either SNP between the NAFLD and control groups, or between the patients with NASH and SS. However, the +45G allele frequency and homozygous variant genotype (GG) were significantly associated with histologically severe fibrosis. Further statistical analysis demonstrated that +45GG was an independent factor of severe fibrosis.

Gupta *et al*^58^ studied two functional polymorphisms of the adiponectin (*ADIPOQ*) gene: +45T/G and −11 377G/C on an Indian population. A cohort of 137 patients with NAFLD, including 113 who underwent liver biopsy, was compared with 250 controls. The case–control study showed both homozygous variants to be significantly more prevalent in the NAFLD cohort, with OR 6.7 (CI 1.5 to 28.6) and OR 5.1 (CI 0.6 to 14) for the −11 377 (CC) and +45(GG) genotypes, respectively. When the biopsied subgroup was considered, patients carrying the −11 377 variant allele correlated with necroinflammatory grade were compared with wild type patients.

##### LEPR

Zain *et al*^59^ investigated the rs1137100 and rs1137101 polymorphisms of the leptin receptor on a multiethnic Malaysian cohort. Results indicated that both polymorphisms were significantly associated with NAFLD, but were not specific to any of the ethnic groups studied. Of the NAFLD cohort, 33 patients had SS and 111 had NASH. Histologically, the rs1137100 GG genotype was observed to be associated with a decreased fibrosis score, and the G allele was protective against fibrosis. The interaction between the *PNPLA3* and leptin receptor polymorphisms was also investigated using a two-locus model, and a significant effect was noted. Aller 2012^60^ investigated the lys656Asn variant of LEPR on 76 obese participants with biopsy-proven NAFLD. There were no significant differences in histological features between genotypes.

Swellam and Hamdy^61^ investigated the leptin receptor SNP rs6700896 in an obese Egyptian cohort of 90 patients with NAFLD and compared with 30 lean, healthy controls. Of the patients with NAFLD, 30 were described as having mild steatosis and 60 had moderate-to-severe steatosis. The mutant allele was not present in the control group, and the mutant genotype was found to be associated with moderate-to-severe steatosis in patients with NAFLD, when compared with those with mild steatosis.

#### Transcription factors

##### STAT3

The *STAT3* gene, encoding signal transducer and activator of transcription 3 (STAT3), is a transcription factor that when activated by growth factors and cytokines, mediates expression of genes involved in a number of different pathways, including cell growth, metabolism, apoptosis and possibly energy homoeostasis(8, 53).

Sookoian *et al*^62^ investigated three polymorphisms in the *STAT3* gene in an Argentinian population: rs2293152, rs6503695 and rs9891119. A cohort of 108 patients with NAFLD, of whom 68 underwent liver biopsy, was compared with 55 healthy controls. In the case–control study, univariate analysis after multiple comparison corrected by permutation tests found significant associations of allelic frequencies of both the rs6503695 and rs9891119 variants with NAFLD. The NAFLD group was histologically divided into fatty liver (not biopsied), fatty liver (biopsied) and definite NASH groups and Spearman rank testing was performed. A significant association was observed with the rs9891119 A allele with increased histological severity, which persisted after BMI was controlled for (AA>AC>CC). However, this result should be interpreted with caution because, although the non-biopsied patients had normal liver function tests (LFTs), histological analysis was not performed on this group. No significant association was observed between *STAT3* variants and inflammation or fibrosis.

##### CLOCK

The *CLOCK* gene encodes a transcription factor involved in regulation of circadian rhythm and metabolism, and variants in *CLOCK* are thought to influence both behaviour and obesity.^9^ Sookoian *et al*^63^ studied six variants of the *CLOCK* candidate gene in an Argentinian population. A cohort of 136 patients with NAFLD was compared with 64 controls; 91 patients were biopsied and 56 were diagnosed with NASH. Univariate analysis showed that both rs11932595 and rs6843722 SNPs were significantly associated with NAFLD compared with control. *CLOCK* variant combinations were analysed using multimarker analysis, some of which showed association, but these did not retain significance with correction for multiple testing. Further omnibus analysis including the six SNPs did show significant results between NAFLD and control, but no individual markers contributed significantly to this. When histology was considered, the rs1554483, rs6843722 and rs6850524 were all significantly associated with disease severity, as were (rs11932595/rs6850524 GC) and (rs4580704/rs6843722 CA) haplotypes, although this should be interpreted with caution because again the entire NAFLD cohort did not undergo biopsy. Within the NASH subgroup, a significant association with overall fibrosis score was observed with rs1554483, rs6843722 and rs4864548.

##### MTHFR

The enzyme methylenetetrahydrofolate reductase (MTHFR) catalyses 5′10-methylenetetrahydrofolate reduction, producing 5′ methyltetrahydrofolate, which is further involved in the formation of methionine from homocysteine.^64^ Methionine is an important methyl donor utilised during the epigenetic process of DNA methylation, and mutations in *MTHFR* have been associated with a number of disease processes.^64^

Serin *et al*^65^ evaluated the *MTHFR* C677T polymorphism in 53 Turkish patients with NAFLD, with 23 patients with NASH, defined by a NAS^3^ of ≥5. The distribution of the polymorphism was found not to be significant between controls (n=282) and NAFLD, or between NAFLD and NASH. Sazci *et al*^64^ also investigated the relationship between polymorphisms of *MTHFR* and NASH in a Turkish population. The two SNPs investigated—C677T and A1928C—were compared between 57 patients with histological NASH, with 324 healthy population controls. Results indicated A1298C was significantly associated with NASH, with the wild type AA being protective against NASH, but there was no significant association between NASH and 677T. The study also looked at compound genotypes and observed that the CC/AC in men and CC/CC genotypes in women were associated with NASH.

Assy *et al*^66^ evaluated thrombotic risk factors, including prothrombin, factor V Leiden and MTHFR mutations. Comparisons were made between 15 patients with fatty liver, 15 with NASH and 15 with chronic viral hepatitis. No significant differences were observed; however, only one variant allele was found within the groups studied.

##### KLF6

The Kruppel-like factor 6 encoded by *KLF6* on chromosome 10 is a zinc finger transcription factor which functions as a tumour suppressor, and polymorphisms have been implicated in various cancer types.^9^ Miele *et al*^67^ investigated the Kruppel-like factor 6 rs37508611 IVSI-27 G>A polymorphism on two cohorts of 306 UK and 109 Italian patients with biopsy-proven NAFLD. In the combined and UK cohorts, a significant reduction in fibrosis score was seen in heterozygous patients compared with homozygous wild type, but results did not reach statistical significance in the Italian cohort, possibly due to the reduced sample size. Logistic regression analysis showed the wild type genotype was a significant independent risk fact for the presence of fibrosis, with (OR 2.76, CI 1.295 to 5.908). This study also investigated inheritance patterns in 71 Italian family trios, which indicated a preferential transmission of the wild type allele.

### Hepatic iron accumulation

#### HFE

The *HFE* gene on chromosome 6 encodes a membrane protein that is hypothesised to regulate iron absorption through the interaction of transferrin with its receptor; homozygotes for mutations (C282Y or H63D) in this gene develop hereditary haemochromatosis.^9^

Valenti *et al*^68^ evaluated polymorphisms in *HFE* in an Italian population comprising 134 patients with NAFLD and 291 healthy controls. Within the NAFLD cohort, 67 underwent biopsy, and NASH was found in 42 patients, whereas 25 had SS. The case–control analysis showed that C282Y (but not H63D) was significantly more prevalent in patients with NAFLD compared with controls. There were no significant differences in frequency of either polymorphism between patients with and without NASH, although only a percentage of the patients with NAFLD were histologically assessed. The same group^69^ studied the *HFE* C282Y and H63D mutations in a larger Italian population. The study compared a cohort of 587 histologically diagnosed patients with NAFLD to 184 healthy controls. The study found the frequency of variant alleles was not significant. It was noted that hepatocellular iron deposition was an independent risk factor for moderate-to-severe fibrosis, but no significant association was found between *HFE* genotype and severity of fibrosis.

Deguti *et al*^70^ evaluated 32 Brazilian patients with histological NASH and found no evidence of association between either C282Y or H63D variant, *HFE* mutations with histological injury and did not observe significant iron deposition. Zamin *et al*^71^ also studied the effects of these *HFE* variants on histological features. A cohort of 29 Brazilian patients with NASH was compared with 20 healthy controls and 20 patients with hepatitis C. The study found the prevalence of the mutation was similar between groups, with no significant association between mutation and fibrosis score or other histological characteristics. An association between liver iron overload and mutation was noted, but this did not correlate with fibrosis score.

Raszeja-Wyszomirska *et al*^72^ studied the effects of C282Y and H63D *HFE* mutations on a Polish cohort of 62 biopsy-proven patients with NAFLD. The authors scored patients on fibrosis as described and compared those with no or mild fibrosis (score 0–2, n=48) against those with advanced fibrosis (score 3–4, n=14). It was observed that in cases with severe fibrosis, the variant H63D allele was associated with increased serum levels of total cholesterol and LDL cholesterol, but no other associations between histological disease severities were apparent.

Nelson *et al*^73^ characterised the C282Y and H63D *HFE* SNPs in 786 participants in the NASH CRN cohort. The relationship between genotype and histological features, including steatosis, inflammation, hepatocyte ballooning, fibrosis stage and NAS,^3^ was evaluated. It was found that participants with the C282Y mutant allele were more likely to have increased iron deposition and hepatocellular pattern, but H63D was not associated with increased hepatic iron accumulation. Both *HFE* SNPs were shown to be associated with an increased steatosis grade when compared with wild type, and H63D allele was also associated with an increase in NAS. A significant reduction in hepatocyte ballooning and definitive NASH associated with the C282Y SNP was also observed; however, there was no association between *HFE* SNPs and fibrosis stage. The authors concluded that the H63D allele was an independent risk factor for the highest grade steatosis, lobular inflammation and NAS ≥5, and definitive diagnosis of NASH.

#### TMPRSS6

The *TMPRSS6* gene encodes transmembrane protease, serine 6, a cell surface enzyme involved in liver matrix remodelling.^9^ The encode enzyme also cleaves a co-receptor necessary for the transcription of hepcidin; therefore, mutations in this gene can affect cellular iron status.^74^

Valenti *et al*^74^ compared the frequency of the *TMPRSS6* AV736 polymorphism in a cohort of 216 Italian patients against a 271 population control. The V/V genotype was observed to be associated with reduced hepatic iron and less severe hepatocyte ballooning, but other histological characteristics of NASH were similar. The study concluded that *TMPRSS6* is not a risk factor for the development of NAFLD.

### Profibrogenic factors

Angiotensin II acts through two receptors to mediate a number of biological effects including vasoconstriction. The angiotensin II receptor 1, encode by *AGTR1* on chromosome 3, is a receptor expressed in the liver by stellate cells that initiates a response to tissue injury.^9^ ^75^ Receptor activation is thought to promote profibrogenic cytokines including transforming growth factor β 1 (TGFB1) which may also have a role in lipid accumulation,^76^ and *SERPINE1* inhibits enzymes involved in fibrinolysis.^9^ Variants in these genes have been investigated as potential risk factors for NAFLD susceptibility.

Yoneda *et al*^77^ performed a case–control study of 167 patients with NAFLD (61 with SS and 106 with NASH) and 435 healthy controls to investigate 12 SNPs of *AGTR1*. It was found that five SNPs were significantly associated with NAFLD when compared with the control group, with rs3772622 having the strongest association. When the SS and NASH groups were compared, the rs3772622 polymorphism was also found to be associated with an increased fibrosis index, with an additive effect.

Zain *et al*^75^ investigated polymorphisms of *AGTR1* in a mixed ethnicity Malaysian cohort of 144 patients with NAFLD compared with 198 healthy population controls. A protective effect against NAFLD was observed in the Indian ethnic subgroup compared with controls, but no other significant differences were noted. There were also no significant associations when patients with NASH were compared with those with SS; however, the GG genotype of rs772622 was shown to be associated with increased severity of histological fibrosis. The interaction between the *PNPLA3* rs738409 variant and *AGTR1* was also analysed; an association with NAFLD susceptibility was observed between the *PNPLA3* variant with both the rs772627 and rs3772630 polymorphisms.

Dixon *et al*^76^ analysed liver biopsy samples from 105 obese Australian patients and evaluated *TGFB1* and *AT* polymorphisms, using reported population data as controls. Of the patients, 103 were diagnosed as having steatosis, and 36 of these also had NASH. The study found no significant association between the polymorphisms investigated separately and presence of fibrosis; however, a combination of the two homozygous mutant genotypes was found to be an independent predictor of advanced fibrosis.

#### SERPINE 1

Espino *et al*^78^ compared the 4G/5G variant of *SERPINE1 (PAI-1)* in 50 obese Chilean patients undergoing bariatric surgery and 71 controls. Within the obese patient group, 21 were histologically normal, 9 had steatosis and 20 met criteria for NASH. No differences in variant frequency were observed between patients and controls, or those with and without NASH-related fibrosis.

### Glucose homoeostasis

#### INSR

The insulin receptor is a tyrosine kinase membrane receptor involved in glucose homoeostasis. The protein encoded by *ENPP1* on chromosome 6 is a cell membrane protein that may be involved in insulin sensitivity and *IRS-1* encodes a substrate involved in the insulin receptor pathway.^9^

Dongiovanni *et al*^79^ evaluated the effect of polymorphisms *ENPP1* rs1044498 and *IRS-1* rs1801278 in a European cohort. A case–control study of 240 Italian patients with NAFLD and healthy population controls showed no significance. Histological features were assessed on a cohort of 702 Italian patients; both polymorphisms were associated with increased fibrosis and the rs1044498 variant allele of *ENPP1* was associated with fibrosis severity.

#### GCKR

*GCKR* encodes glucokinase regulator, a protein that modulates the activity of the enzyme glucokinase in liver cells by reversibly binding to form an inactive complex.^9^ Tan *et al*^80^ investigated the *GCKR* rs780094 (C>T) and rs1260326 (C>T) SNPs on a mixed ethnicity Malaysian cohort of 144 patients with NAFLD and 198 controls. The variant T allele frequencies of each SNP was found to be more prevalent in patients with both NAFLD and NASH when compared with controls; however, when the ethnic subgroups were considered, the relationship was only significant in the Indian population, not those of Chinese or Malay ethnicity. Owing to the mixed ethnicity of this cohort, the results should be interpreted with caution.

#### TCF7L2

Transcription factor 7-like 2 is a transcription factor expressed in T cells containing a high mobility group box that is thought to be involved in glucose homoeostasis.^9^ Musso *et al*^81^ investigated the rs7903146 variant of the *TCF7L2* gene on an Italian population. A total of 78 patients with NAFLD, 34 of whom were biopsied and diagnosed with NASH, were compared with 156 healthy population controls. It was found that the wild type genotype was less frequent in patients with NAFLD compared with controls. The study reports that severe hepatic steatosis was predicted by *TCF* (OR 2.2, CI 1.7 to 4.9) and grade 3 fibrosis was predicted by *TCF* (OR 2.0, CI 1.7 to 4.3); however, the analysis of histological characteristics is limited due to small sample size and because all patients had NASH.

### Neurological

The cannabinoid receptors are G protein coupled receptors expressed in the brain that mediate the neurological effects of cannabinoid substances through inhibition of adenylyl cyclase when activated.^9^ The *NCAN* gene on chromosome 19 encodes the core protein of neurocan, a chondroitin sulfate proteoglycan component of the extracellular matrix, primarily expressed in the nervous system and considered to be involved in cell adhesion and migration.^9^ ^23^

### Cannabinoid receptor

Aller *et al*^82^ evaluated the influence of the cannabinoid receptor 1 gene rs1049353 variant on histological features of NAFLD on a cohort of 70 Spanish patients. The study found that significantly fewer patients with the variant A allele had histological grade of 4 or more, when compared with homozygous wild type patients, but fibrosis was not significantly different.

Rossi *et al*^83^ investigated the effect of the Q63R variant of cannabinoid receptor 2 on histological severity of NAFLD in a cohort of 118 paediatric Italian patients. The variant was found to be strongly associated with degree of inflammation, but not with other histological characteristics of steatosis, fibrosis or ballooning. However, the relationship with inflammation was not significant after adjusting for the *PNPLA3* variant, which had previously been shown to be significant in this cohort.^16^

#### NCAN

Gorden *et al*^23^ studied a cohort of 1092 US patients undergoing bariatric surgery, as described above. The variant in *NCAN* was significantly associated with increased histological features of steatosis, lobular inflammation and perivenular fibrosis, and trended towards increased ballooning. However, it is unclear whether these associations are independent of the association with the *PNPLA3* rs738409 shown to be significantly associated with steatosis and ballooning in the same study.

### Miscellaneous

#### TM6SF2

*TM6SF2* encodes a transmembrane protein of unknown function;^9^ the rs58542926 SNP in *TM6SF2* is in strong linkage disequilibrium with the rs2228603 *NCAN* variant discussed above, and associations attributed to *NCAN* may relate instead to this variant.^84^

Liu *et al*^84^ studied the effect of the rs58542926 variant of *TM6SF2* on two cohorts with histologically characterised NAFLD. Quantitative analysis was performed within a discovery cohort of 349 UK patients with NAFLD, and results were validated using a cohort of 725 European Caucasian patients. Within the NAFLD discovery cohort, minor allele was significantly more frequent than the reference population, OR 1.72 (CI 1.16 to 2.57) and OR 4.84 (CI 1.01 to 22.9) for heterozygous and homozygous variants, respectively, when compared with healthy control group (n=379).

Histological characteristics were further investigated: degree of steatosis was not significantly associated with either rs2228603 in *NCAN* or rs58542926 in *TM6SF2*, in either cohort, although a trend was observed when the cohorts were combined. The authors suggest a possible relationship with a small effect size, which was supported by an association which was noted when the combined group was categorised into mild or pronounced steatosis. Severity of steatohepatits, assessed by characteristics of necroinflammation and hepatocyte ballooning, was associated with the *TM6SF2* variant within the discovery cohort, but was not replicated in the validation or combined cohorts.

Importantly, the variants of *NCAN* and *TM6SF2* were both significantly associated with the stage of histological fibrosis. On further investigation, conditioning the results on *TM6SF2* negated the effect of the *NCAN* variant, indicating *TM6SF2* was responsible for the association relationship. This relationship was replicated in the validation and combined cohorts, and was independent of confounding factors including age, gender, BMI, presence of diabetes and *PNPLA3* phenotype. When mild fibrosis was compared with advanced fibrosis in the combined cohort, each minor allele was associated with OR 1.88 (CI 1.41 to 2.50). The study also compared 99 UK patients with NAFLD-related HCC to the combined cohort. Although the homozygous variant of *TM6SF2* was found associated with an increased risk of HCC in univariate analysis, this did not persist in multivariate analysis adjusted for confounding factors.

Since this study, two other studies have investigated the role of this polymorphism on histologically proven NAFLD: Dongiovanni *et al*^85^ studied a large, combined cohort of adult and paediatric European patients undergoing liver biopsy for hepatology or bariatric purposes. Of these, 112 patients had no fatty liver disease and served as the control group, 412 had SS and 677 fulfilled histological NASH diagnosis. The results showed the variant to be associated with severity of all histological characteristics (steatosis, necroinflammation, ballooning and fibrosis stage), and this persisted after adjustment for age, gender, BMI, diabetes, recruitment and *PNPLA3* genotype. The variant was associated with NASH compared with SS (OR 1.84, CI 1.23 to 2.79) and stage 3–4 fibrosis (OR 2.08, CI 1.20 to 3.55), although association with fibrosis did not remain after adjustment for NASH.

Sookoian *et al*^86^ performed a case–control study investigating the role of on *TM6SF2* in an Argentinian population of 226 patients with histological NAFLD. In comparison to a control group of 135 individuals with no ultrasonographic evidence of fatty liver, the rs58542926 variant was found to be significantly associated with fatty liver (OR 1.37, CI 1.02 to 1.84); however, this relationship did not persist after adjusting for age, BMI and rs738409 in *PNPLA3*. The NAFLD group was subdivided into 96 patients with SS and 130 with NASH; the T allele was found to be associated with disease progression when the control group was included in the analysis; however, this was not significant when the SS group was compared with NASH. Within the patients with NAFLD, degree of steatosis was found to be significantly associated with this variant, independently of *PNPLA3* genotype, age, sex and BMI. In contrast, no associations were observed with other histological characteristics of lobular inflammation, ballooning or fibrosis stage (F1–2 vs F3–4), and there was no association with increased NAS (defined as >5).

#### SPINK1

The *SPINK 1* gene encodes serine peptidase inhibitor, Kazal type 1.^9^ This enzyme inhibits trypsin, and is a component of pancreatic juice, secreted by pancreatic acinar cells; mutations result in familial calcific pancreatitis.^9^ Oruc *et al*^87^ evaluated the *SPINK1* N34S P555 polymorphism in 50 Turkish patients with NAFLD and 44 controls. Of the NAFLD group, 30 patients had SS and 20 had NASH. No significant association was found between the *SPINK1* variant with NAFLD or histological NASH.

##### ABCB11 and NR1H4

Both the *ABCB11* and *NR1H4* genes encode proteins involved in bile processing: ATP binding cassette transporter encoded by *ABCB11* is a membrane-bound transport protein that exports of bile salts into canaliculi, whereas *NR1H4* encodes a nuclear receptor activated by bile acids that regulates their synthesis and transport.^9^ Iwata *et al*^88^ evaluated the *ABCB11* 1331C allele and *NRIH4* variants, comparing UK patients—358 with NAFLD, 206 with chronic hepatitis C and 110 controls undergoing biopsy for metastatic disease. There was no association found with NAFLD or fibrosis for either gene.

##### CYP2E1 and NR1I2

*CYP2E1* encodes a cytochrome P450 enzyme involved in metabolism of endogenous and exogenous compounds, and *NR1I2* encodes the pregnane X receptor, a transcriptional regulator of *CYP3A4.*^9^

Varela *et al*^89^ studied cytochrome *P450 2E1* variants on 48 obese Chilean women undergoing bariatric surgery. Patients were biopsied and assessed: 15 were diagnosed with NASH, 17 had SS and 13 were histologically normal. The *CYP4502E1* polymorphisms Dra1 and Ras1/Pst1 were investigated and it was found that the heterozygous and homozygous variant genotypes of Dra1 increased the risk of both NASH and steatosis when compared with control. The Rsa/Pst variant genotypes also increased the risk of NASH compared with control, although it should be noted that the sample sizes utilised in this study were small.

Sookoian *et al*^90^ explored eight SNPs associated with *NR1I2*; only rs7643645 was significantly associated with NAFLD compared with control, with a recessive model for the homozygous variant with OR 3.292 (CI 1.398 to 7.752). Of 188 patients with NAFLD, 113 underwent liver biopsy; 46 had SS and 67 had NASH. Participants were divided into control, fatty liver with normal LFTs, SS and NASH groups to investigate disease severity, noting associations with rs7643645 and rs2461823.

##### FABP2 and SLC27A5

The fatty acid-binding protein 2 and fatty acid transport protein 5, encoded by *FABP2 and SLC27A5*, respectively, are involved in transport and processing of long chain fatty acids.^9^ Auinger *et al*^91^ explored the rs56225452 variant of *FATP5* (*SLC27A5*) on a cohort of 103 male patients with NAFLD. No difference between genotypes was observed in features of steatosis, fibrosis or necroinflammatory grade, although an association was observed with steatosis degree and BMI within variant allele carriers. Aller *et al*^92^ evaluated the effect of ala54thr of *FABP2* on 30 patients with NAFLD, but no differences between histological features were observed.

### Genome-wide association studies

GWASs aim to identify genetic susceptibility components that might predispose individuals to developing NAFLD and NASH. A total of five reports of GWASs have been performed on the NASH CRN and Japanese populations.

Chalasani *et al*^93^ performed a GWAS analysing 324 623 SNPs on 236 female patients in the US NASH CRN cohort, diagnosed using the NASH CRN scoring system. Results showed that *FDFT1* variant rs2645424 was associated with NAS, fibrosis was associated with rs343062 on chromosome 7, and lobular inflammation was associated with SNPs in *LTBP3*, *EFCAB4B* and *COLI3A1*.

Speliotes *et al*^94^ performed primary GWAS testing on a large cohort of patients with CT assessed NAFLD, from which 46 SNPs were selected for further analysis in patients with histologically determined NAFLD. A total of 592 patients within the NASH CRN cohort were compared with 1405 US and European healthy patients enrolled in the MIGen study. The rs738409 in *PNPLA3* and rs2228603 in *NCAN* were found to be significantly associated with histological NAFLD, with ORs of 3.26 and 1.65, respectively. The rs4240624 in *PPP1R3B* was not associated with histological NAFLD; however, rs780094 in *GCKR* (OR 1.45) and rs12137855 near *LYPLAL1* (OR 1.37) were also significantly associated with NAFLD. The MIGen cohort was used to control for cardiovascular disease status. Therefore, to ensure that the use of this control group was not influential, the five variants (*PNPLA3, NCAN, GCKR, LYLPAL1* and *PPP1R3B*) were compared between the NASH CRN cohort and 3212 healthy participants within the Illuminata Control Database (iCONT). The results of this were comparable to those from the NASH CRN/MIGen comparison.

Kawaguchi *et al*^11^ performed a GWAS of 484751 SNPs plus *PNPLA3* rs738409 on a Japanese population. Initially, 529 patients with NAFLD were compared with 942 healthy controls, and significance was observed for six markers in *PNPLA3* including rs738409, which demonstrated the strongest association. The NAFLD group was further divided into four subgroups based on histological class, and genotype distributions were analysed in a pairwise manner. Matteoni^95^ class 4 was found to be significantly different from the other three subgroups, and associated with the rs738409 variant. Histological characteristics of Brunt grade and stage,^96^ fat deposition, iron deposition were associated with the variant G allele; however, of these only iron deposition remained significant after Bonferroni correction and adjustment for Matteoni type. The authors propose that NASH (assessed as Matteoni type 4) is a clinically and genetically different condition. Of the other polymorphisms investigated, no association was seen with variants in *LYPLAL1* or *NCAN* genes, but rs780094 in *GCKR* showed borderline association.

Kitamoto *et al*^97^ performed a pilot GWAS on 392 Japanese patients with NAFLD (345 NASH, 47 SS). Initially, 261 540 genes were analysed and compared with a group of 934 healthy controls. Subsequently, 56 SNPs were further analysed in a replication study, comprised of 172 patients with NAFLD (97 NASH, 4 SS, 71 not biopsied) and 1012 healthy controls. Following the replication study, 12 SNPs remained significantly associated following the replication study, and of these, 8 retained significance after conservative Bonferroni correction. These 8 SNPs were all variants close to *PNPLA3* on chromosome 22, and did not retain significance after rs738409 was accounted for, and haplotype analysis suggested that *PNPLA3* was significantly associated with NAFLD pathogenesis. When histological characteristics were assessed, six polymorphisms were associated with histological fibrosis, and nine were associated with lobular inflammation, hepatocyte ballooning and NAS, but these were not significant after correction for multiple testing. When NASH patients were compared with those with SS, the rs5764455 variant in *PARVB* was associated with NASH (OR 2.79).

Vazquez-Chantada *et al*^98^ extracted DNA from liver biopsy samples and tested 92 genes for association with NAFLD in a Spanish pilot study of 69 patients with NAFLD and 217 controls. In total, 1536 SNPs were selected for further analysis and 11 SNPs were found to be associated with NAFLD, 7 of these in the *SLC2A1* gene and 1 close to *SLC2A1*, and variants in *CYP2E1, MTR* and *STK11*. A validation study was then performed on a cohort of 451 European patients with NAFLD and 303 controls, and confirmed that rs4658 and rs841856 in *SLC2A1* were significantly more frequent in the NAFLD group, however no association was seen in the other variants. The study found no significant association with clinical characteristics of NAFLD.

## Conclusion

Variants in many genes have been investigated for association with NAFLD and with histological characteristics and progression to NASH. Of the genes studied, many could have plausible mechanistic roles in NAFLD and NASH pathogenicity, for example, in profibrogenic and inflammatory pathways, regulation of energy, metabolism and lipid processing. Other genes investigated are those that have potential roles in obesity, hypertension and dyslipidaemias.

Only six genes described in this review have been replicated in more than one histologically characterised cohort: *PNPLA3, APOE, SOD2, TNF, TM6SF2* and *GCKR* (highlighted in table 1). The main, consistent finding of the candidate gene studies is the association of *PNPLA3* rs738409 variant with NAFLD susceptibility. Results regarding the association of this polymorphism with histological characteristics and disease severity are inconsistent.

Differences may be due to patient characteristics including environment or ancestry, with findings and the strength of associations differing between populations. Even within a biopsy proven cohort, there are further confounding factors of age, sex, BMI, comorbidities including diabetes, diet, exercise and medication to consider. Methodological factors including the size of patient cohorts and control population(s), the method and criteria used for histological assessment, which are not necessarily comparable, and statistical handling of data may also account for discrepancies.

Liver biopsy is an invasive procedure that can involve complications of infection, haemorrhage and an approximate 1/10 000 mortality.^99^ Therefore, larger cohorts of biopsied patients, such as the NASH CRN, Malaysian cohort and the Italian and UK groups have been used in multiple investigations, which needs to be borne in mind when considering effects of marginal significance.

Various study designs were used to address differing putative gene effects, studies comparing patient cohorts to healthy control groups were used to assess for genetic association with disease. Severity of histological characteristics were compared within patient cohorts, and some studies used control groups with other causes of hepatic fibrosis, such as alcoholic liver disease or hepatitis C to determine whether genetic factors influencing fibrosis were specific to NASH or related to common pathways of fibrosis. It may be that there are multiple genetic factors that come into play, with separate groups of genes influencing NAFLD susceptibility, and different molecular pathways involved steatosis, inflammation and fibrosis of disease progression.

In this review, methods for reporting results were restricted to individual genes; however, a novel method of reporting using PID pathways was utilised by Chen *et al*^6^ (not included in review), which may be useful to identify the molecular pathways involved in the development of NAFLD and progression to NASH. Similarly, as whole genome scanning becomes more time and cost efficient, it is likely that more studies will utilise this technique for investigating genetic factors of a disease.

This review has aimed to independently and systematically summarise the candidate gene studies relating to NAFLD and NASH. Fatty liver is a complex disease, with a spectrum of severity encompassing clinical features of steatosis, inflammation and fibrosis. Only a subgroup of patients with SS progresses to develop NASH and hepatic fibrosis. It is possible that there are multiple genetic components influencing susceptibility through distinct pathways, and that the interaction of these may cause disease progression.

## Supplementary Material

Supplementary Materials
