# Supplementary material for: Systematic review of genetic association studies involving histologically confirmed non-alcoholic fatty liver disease
Source: BMJ Open Gastroenterol. 2015 Feb 17;2(1):e000019. doi: 10.1136/bmjgast-2014-000019 (PMC4599155; doi:10.1136/bmjgast-2014-000019)
Supplement: Supplementary Materials [file bmjgast-2014-000019.html]

Systematic review of genetic association studies involving histologically confirmed non-alcoholic fatty liver disease: BMJ Open Gastroenterology: Vol 0, No 0

Supplementary tables
