# Supplementary material for: Systematic review of genetic association studies involving histologically confirmed non-alcoholic fatty liver disease
Source: BMJ Open Gastroenterol. 2015 Feb 17;2(1):e000019. doi: 10.1136/bmjgast-2014-000019 (PMC4599155; doi:10.1136/bmjgast-2014-000019)
Supplement: Supplementary Materials [file bmjgast-2014-000019supp_tables.pdf]

Supplementary Table 1. Summary of studies included in systematic review

First author, year	Population / Location	Histological method	NAFLD (n)	Control (n)	Study type	Candidate gene(s)
Hotta, 2010	Japanese	Sanyal, 2002[1]<br>Matteoni, 1999[2]<br>Teli, 1995[3]<br>Brunt, 2001[4]	253	578	Association study	*PNPLA3*
Kawaguchi, 2012	Japanese	Matteoni, 1999[2]<br>Brunt, 1999[5]	529	942	Association study	*PNPLA3*
Rotman, 2010	NASH CRN (US)	Brunt, 2009[6]<br>Kleiner, 2005[7]	894 adult<br>223 paediatric	336<br>-	Association study	*PNPLA3*
Sookoian, 2009	Argentinian	Brunt, 1999[5]<br>Kleiner, 2005[7]	172*	94	Association study	*PNPLA3*
Sookoian, 2011					Meta-analysis	*PNPLA3*
Speliotes, 2010	NASH CRN (US)	Kleiner, 2005[7]	592	1405	Association study	*PNPLA3*
Valenti, 2010a	Italian cohort/ UK cohort	Kleiner, 2005[7]	253<br>321	179<br>-	Association study	*PNPLA3*
Valenti, 2010b	Italian - Paediatrics	Kleiner, 2005[7]	149	0	Histological severity	*PNPLA3*
Zain, 2012	Malaysian	Brunt, 2011 [8]<br>Kleiner, 2005[7]	144	198	Association study	*PNPLA3*
Corbin, 2013	NASH CRN (US)	Kleiner, 2005[7]	361	85	Association study	21 genes
Guichelaar, 2013	Obese	Kleiner, 2005[7]	144 obese	-	Histological severity	*PNPLA3*
Verrijken, 2013	Belgian - obese	Brunt, 2011[8]<br>Kleiner, 2005[7]	470* obese	0	Histological severity	*PNPLA3*<br>*APOC3*
Valenti, 2012	Italian	Kleiner, 2005[7]	257	337	Association study	*LPIN1*
Gawrieh, 2012	US	Kleiner, 2005[7]	212	62	Association study	*PPARG*
Rey, 2010	German	Kleiner, 2005[7]	263	259 Healthy<br>100 AFL	Association study	*PPARG*
Sahebkar, 2013					Meta-analysis	*PPARG*
Domenici, 2013	Brazilian	Brunt, 2010 [9]<br>Brunt, 2011[8]	103	103	Association study	*PPARG*<br>*PPARA*
Dongiovanni, 2010	Italian	Kleiner, 2005[7]	202	346	Association study	*PPARG*<br>*PPARA*
Yoneda, 2008	Japanese	Matteoni, 1999[2]<br>Teli, 1995[3]	115	441	Association study	*PPARGC1A*
Valenti, 2011	Italian cohort/ UK cohort	Kleiner, 2005[7]	758	316	Association study	*APOC3*
de Feo, 2012	Italian	Brunt, 1999[5]	310*	422	Association study	*APOE*
Musso, 2009	Italian	Brunt, 2001[4]	78*	156	Association study	*APOE*
Sazci, 2008	Turkish	Kleiner, 2005[7]	57	245	Association study	*APOE*
Musso, 2011	Italian	Brunt, 2001[4]	40	40	Association	*APOE*

					study	*OLR1*<br>*MTP*
Gambino, 2007	Italian	Brunt, 2001[4]	29	29	Association study	*MTP*
Dong, 2007	Japanese	Brunt, 1999[5]	107	150 Healthy<br>100 HCV	Association study	*PEMT*
Carulli, 2009	Italian	Brunt, 2004[10]	114*	79	Association study	*MTP*<br>*PC1*<br>*IL6*
Oliviera, 2010	Brazilian	Kleiner, 2005[7]	139	141	Association study	*MTP*<br>*GCLC*
Namikawa, 2004	Japanese	Brunt, 1999[5]<br>Brunt, 2001[4]	63	150	Association study	*MTP*<br>*SOD*
El-Koofy, 2010	Egyptian paediatrics	Kleiner, 2005[7]	76*	20	Association study	*MTP*<br>*SOD2*
Musso, 2013a	Italian	Kleiner, 2005[7]<br>Brunt, 2001[4]	127*	48	Longitudinal	*SREBFP2*
Musso, 2013b	Italian	Kleiner, 2005[7]<br>Brunt, 2001[4]	161*	51	Longitudinal	*SREBPF1C*
Al-Serri, 2012	Italian cohort/ UK cohort	Brunt, 1999[5]	510	0	Histological severity	*SOD2*
Aller, 2010	Spanish	Brunt, 2001[4]	39	0	Histological severity	*UCP3*
Brun, 2006	Italian	Not described	28	52	Association study	*CD14*<br>*TLR4*
Nozaki, 2004	Japanese	Brunt, 1999[5]<br>Brunt, 2001[4]	63	100	Association study	*IL1B*<br>*ADRB3*
Petta, 2012	Sicilian cohort	Kleiner, 2005[7]	165	0	Histological severity	*IL28B*
Akyildiz, 2010	Turkish	Kleiner, 2005[7]	91	104	Association study	*MIF*
Aller, 2010	Spanish	Brunt, 2001[4]	66	213	Association study	*TNF*
Chowdhury, 2013	Indian	Kleiner, 2005[7]	29	0	Association study	*TNF*
Hu, 2013	Chinese	Kleiner, 2005[7]	189*	138	Association study	*TNF*
Tokushige, 2007	Japanese	Neuschwander-Tetri, 2003[11]<br>Brunt, 1999[5]<br>Brunt, 2001[4]<br>Kleiner, 2005[7]	102	100	Association study	*TNF*
Valenti, 2002	Italian	Sheth, 1997[12]<br>Brunt, 2001[4]	99*	172	Association study	*TNF*
Yang, 2012	Korean - paediatric, obese	Brunt, 1999[5]<br>Kleiner, 2005[7]	50	61	Association study	*TNF*
Wong, 2008	Chinese	Brunt, 1999[5]	79	40	Association study	*TNF*<br>*ADIPOQ*
Gupta, 2012	Indian	Brunt, 2009[13]<br>Kleiner, 2005[7]	137*	250	Association study	*ADIPOQ*
Tokushige, 2009	Japanese	Brunt, 1999[5]<br>Brunt, 2001[4]<br>Kleiner, 2005[7]	119	115	Association study	*ADIPOQ*
Aller, 2011	Spanish	Brunt, 2001[4]	76	0	Histological severity	*LEPR*
Swellam, 2012	Egyptian	Not described	90	30	Association study	*LEPR*

Zain, 2013	Malaysian	Brunt, 2011[8]<br>Kleiner, 2005[7]	144	198	Association study	*LEPR*
Sookoian, 2007	Argentinian	Brunt, 1999[5]	136*	64	Association study	*CLOCK*
Miele, 2008	Italian cohort/ UK cohort	Brunt, 1999[5]	415	0	Histological severity	*KLF6*
Sazci, 2008	Turkish	Brunt, 1999[5]<br>Kleiner, 2005[7]<br>Brunt, 2004[10]	57	324	Association study	*MTFTR*
Serin, 2007	Turkish	Kleiner, 2005[7]	53	282	Association study	*MTFTR*
Assy, 2005	Israeli	Brunt, 1999[5]	30	10 Healthy<br>15 HCV	Association study	*MTHFR*<br>*Prothrombin*<br>*Factor V Leiden*
Sookoian, 2008	Argentinian	Brunt, 1999[5]	108*	55	Association study	*STAT3*
Deguti, 2003	Brazilian	Not described	32	0	Histological severity	*HFE*
Nelson, 2012	NASH CRN (US)	Kleiner, 2005[7]	786	0	Histological severity	*HFE*
Raszeja-Wyszomirska, 2010	Polish	Not described	62	0	Histological severity	*HFE*
Valenti, 2003	Italian	Sheth, 1997[12]	134*	291	Association study	*HFE*
Valenti, 2008	Italian	Kleiner, 2005[7]	587	184	Association study	*HFE*
Zamin, 2006	Brazilian	Brunt, 1999[5]	29	20 Healthy<br>20 HCV	Association study	*HFE*
Valenti, 2012b	Italian	Kleiner, 2005[7]	216	271	Association study	*TMPRSS6*
Zain, 2013	Malaysian	Brunt, 2011[8]<br>Kleiner, 2005[7]	144	198	Association study	*AGTR1*
Yoneda, 2009	Japanese	Matteoni, 1999[2]<br>Teli, 1995[3]<br>Brunt, 2001[4]	167	435	Association study	*AT2*
Espino, 2011	Mexican	Kleiner, 2005[7]	29	21	Association study	*SERPINE1*
Dixon, 2003	Australia	Brunt, 1999[5]<br>Lee, 1998 [14]<br>Lee, 1995[15]	105	0	Histological severity	*TGFB1*<br>*AT6*
Dongiovanni, 2010	Italian cohort/ UK cohort	Kleiner, 2005[7]	702	310	Association study	*ENPP1*<br>*IRA1*<br>*PC1*
Tan, 2013	Malaysian	Brunt, 1999[5]<br>Kleiner, 2005[7]	144	198	Association study	*GCKR*
Aller, 2012	Caucasian (Spanish)	Brunt, 2001[4]	70	0	Histological severity	*CBR1*
Rossi, 2012	Italian - Paediatrics	Kleiner, 2005[7]	118	0	Histological severity	*CBR2*
Gorden, 2013	US	Kleiner, 2005[7]	748	344	Association study	*NCAN*
Lui, 2014	UK<br>European	Bedossa, 2014[16]	349<br>725	265	Association study	*TM6SF2*<br>*NCAN*
Dongiovanni, 2014	European	Kleiner, 2005[7]	1089	112	Association study	*TM6SF2*

Sookoian, 2014	Argentinian	Kleiner, 2005[7]<br>Brunt, 2011[8]	226	135	Association study	*TM6SF2*
Oruc, 2009	Turkish	Not described	50	44	Association study	*SPINK1*
Iwata, 2011	UK	Brunt, 1999[5]	358	110 Control**<br>206 HCV	Association study	*ABCB11*<br>*NRIH4*
Varela, 2008	Chilean	Not described	32	13	Association study	*CYP2E1*
Sookoian, 2010	Argentinian	Brunt, 1999[5]	188*	102	Association study	*NR1I2*
Aller, 2009	Spanish	Brunt, 2001[4]	30	0	Histological severity	*FABP2*
Auinger, 2010	Italian	Kleiner, 2005[7]	103	0	Histological severity	*SLC27A5*
Chalassni, 2010	NASH CRN (US)	Kleiner, 2005[7]	236	0	GWAS	
Kitamoto, 2013	Japanese	Brunt, 2001[4]<br>Kleiner, 2005[7]<br>Matteoni, 1999[2]<br>Teli, 1995[3]	392	934	GWAS	
Speliotes, 2011	NASH CRN (US)	Kleiner, 2005[7]	592	1405	GWAS	
Vazquez-Chantada, 2013	Spanish<br>European	Brunt, 1999[5]<br>Kleiner, 2005[7]	69<br>451	217<br>303	GWAS	

N.B Association studies include case-control association and histological association
HCV: Chronic hepatitis C patients; AFL: alcoholic fatty liver patients
***Total NAFLD group, only partially histologically diagnosed**
****Control group undergoing resection for hepatic metastases of non-liver primary cancer**

Supplementary Table 2. Summary of candidate genes and polymorphisms included in systematic review

Gene symbol [17]	Description of encoded protein [18]	Polymorphisms [19]	Genomic alteration (g.) [20]	Type [19, 20]	Protein change [20]	Global MAF [19]
ABCB11	Membrane transporter, bile salt export	rs2287622	A>C, A>G, A>T	Missense	Val444Gly, Val444Ala, Val444Asp	A=0.4059/884
ABCB4	Membrane transporter, may be involved in hepatic biliary phospholipid export	rs31672	C>T	Intron variant		C=0.3388/738
ADIPOQ	Adiponectin, an adipocytokine	rs2241766	T>G	Synonymous		G=0.1446/314
		rs17300539	G>A	Upstream gene variant		A=0.0399/86
		rs1501299	G>T	Intron variant		T=0.3200/696
		rs2241766	T>G	Synonymous		G=0.1446/314
		rs266729	C>G	Upstream gene variant		G=0.2507/546
ADRB3	G-protein coupled adrenergic receptor, involved in regulation of lipolysis and thermogenesis in adipose tissue	rs4994	A>G	Missense	Trp64Arg	G=0.1001/218
AGTR1	Angiotensin II receptor, mediates cardiovascular effects of RAAS	rs772627	T>A	Intergenic variant		T=0.4904/1067
		rs3772622	T>C	Intron variant		C=0.3682/802
		rs3772630	T>C	Intron variant		C=0.4316/940
APOC3	VLDL component, inhibits hepatic lipase	rs2854116	C>T	Upstream gene variant		T=0.4940/1075
		rs2854117	T>C	Upstream gene variant		T=0.4550/991
APOE3	Chylomicron component, mediates hepatic uptake and clearance	CM890009 (e3)	T>C	Missense	Arg160Cys	Not known
		rs429358 (e4)	T>C	Missense	Cys156Arg Pathogenic	C=0.1492/324
CHDH	Enzyme choline dehydrogenase	rs4563403	C>T	3' variant		T=0.2374/517
Chromosome 7		rs343064	C>T	Upstream variant		T=0.3609/786
CHUK	Serine kinase involved in NF-kappa-B signalling	rs11591741	G>C	Intron variant		C=0.2277/496
		rs11597086	A>C	Intron variant		C=0.2227/484
CLOCK	Transcription factor involved in circadian rhythm regulation	rs6850524	C>G	Intron variant		C=0.4293/935
		rs11932595	A>G	Intron variant		G=0.3264/710
		rs1554483	C>G	Intron variant		G=0.3866/841

		rs4580704	G>C	Intron variant		G=0.2961/644
		rs4864548	G>A	Non-coding exon variant		A=0.3861/840
		rs6843722	A>C	Intron variant		C=0.3613/787
CNR1	GPCR in brain, mediates CNS effects of cannabis components	rs1049353	C>T	Synonymous		T=0.1373/299
CNR2	GPCR in brain, mediates CNS effects of cannabis components	rs2501432	T>C	Missense	Gln63Arg	T=0.4835/1052
COL13A1	Nonfibrillar type collagen	rs1227756	G>A	Intron variant		A=0.3916/853
CPN1	Plasma metallo-protease	rs11597390	G>A	Regulatory region variant		A=0.2534/552
CYP2E1	Metabolic liver enzyme	rs6413432 (DraI)	T>A	Intron variant	Pathogenic - affects enzyme activity	A=0.1547/336
		rs2031920 (RasI)	C>T	Upstream variant	Pathogenic - altered transcription	T=0.0895/195
		rs3813867 (PstI)	G>C	Upstream variant	Pathogenic - altered transcription	C=0.1006/219
EFCAB4B	Interacts with calcium in T lymphocytes, regulating the calcium release activating channel	Rs887304	T>C	3 prime UTR variant		T=0.1543/336
ENPP1	Transmembrane glycoprotein, cleaves numerous substrates and involved in tissue calcification	rs1044498	A>C	Missense	Lys173Gln	C=0.2925/636
FABP2	Intracellular protein involved in long chain fatty acid processing	rs1799883	T>G	Missense	ala54thr	T=0.2521/548
FATP5	Involved in hepatic uptake of fatty acids and bile metabolism	rs56225452	C>T	Intron, downstream and upstream variant		T=0.1621/352
FDFT1	Enzyme, in cholesterol biosynthesis pathway	rs2645424	A>G	Intron variant		G=0.4665/1015
GCKR	Inhibits glucokinase enzyme in the liver	rs780094	T>C	Intron variant		T=0.3857/840
		rs1260326	T>C	Missense	Leu446Pro	T=0.3848/838
HFE	Membrane protein, thought to have a role regulating iron absorption	rs1800562	G>A	Missense	Cys282Tyr	A=0.0197/43
		rs1799945	C>G	Missense	His63Asp	G=0.0836/181
IFNL3 (IL28B)	Proinflammatory cytokine	rs8099917	T>G	Intron variant		G=0.1382/300

		rs12979860	C>T	Intron variant		T=0.3388/738
IL1B	Proinflammatory cytokine	rs16944	A>G	Upstream gene variant		A=0.4651/1013
IL6	Proinflammatory cytokine	rs1800795	C>G	Upstream gene variant		C=0.1850/402
IRS1	Involved in pathways activated by insulin	rs1801278	C>A, C>T, C>G	Missense	Gly971Trp, Gly971Arg	T=0.0537/116
KLF6	Tumour suppressing transcription factor	rs3750861	C>T	Intron variant		T=0.0606/132
LEPR	Regulates fat metabolism	rs6700896	C>T	Intron variant		C=0.4591/1000
		rs1137100	A>G	Missense	Lys109Arg	G=0.3852/839
		rs1137101	A>G	Missense	Gln223Arg	A=0.4105/893
LPIN1	Required for adipocyte differentiation and transcriptional cofactor involved in lipid metabolism	rs13412852	C>T	Intron variant		T=0.2461/535
LTBP3	Interacts with TGF-beta; possible role in TGF-beta translocation and targeting	Rs6591182	T>G	Missense	Val538Gly	G=0.4316/940
LYPLAL1	Lysophospoholipase enzyme activity	rs12137855	C>T	Downstream gene variant		T=0.1648/358
MIF	Proinflammatory cytokine	rs755622	G>C	Upstream gene variant		C=0.2723/592
MTHFR	Enzyme that catalyses reaction producing a co-substrate in homocysteine remethylation	rs7525338	C>T	Intron variant		T=0.0161/35
NCAN	Proteoglycan, involved in cell adhesion and migration	Rs2228603	C>T	Missense	Pro92Ser	T=0.0468/102
NR1I2	Ligand activated transcription factor, involved in cytochrome P450 CYP3A4 regulation	rs1248820	T>C	Upstream gene variant		C=0.4215/918
		rs1054191	A>G	3 prime UTR variant		A=0.1171/255
		rs2461823	A>G	Intron variant		T=0.3871/842
		rs2472671	C>T	Intron variant		C=0.2153/468
		rs3814055	C>T	Upstream variant, 5' UTR variant		T=0.3242/706
		rs3814057	A>C	3 prime UTR variant		C=0.3255/708
		rs6785049	G>A	Intron variant		A=0.4444/967
		rs7643645	A>G	Intron variant		G=0.3209/699
PARVB	Role in cytoskeleton and cell adhesion, binds actin	rs5764455	A>G	Intron variant		A=0.4164/906
PEMT	Liver enzyme, catalyses production of	rs7946	C>T	Missense	Val175Met	T=0.4614/100

	phosphatidylcholine required for VLDL secretion					5
		rs13342397	T>C	intron variant		C=0.10 19/221
		rs8068641	A>G	intron variant		G=0.21 58/469
		rs936108	C>T	intron variant		T=0.41 64/906
PNPLA3	Triglyceride lipase enzyme expressed by adipocytes	rs738409	C>G	Missense	Ile148Val	G=0.28 42/619
		rs2281135	G>A	intron variant		A=0.27 23/592
		rs2294918	A>G	Missense	Lys434Glu	A=0.22 96/499
PPARA	Transcription factor, stimulates fatty acid catabolism in the liver	rs1800206	C>G	Missense	Leu162Val	G=0.02 48/54
PPARG	Transcription factor, stimulates adipocyte differentiation and fatty acid storage	rs3856806	C>T	Synonymous		T=0.11 85/257
		rs1801282	C>G	Missense	Pro12Ala	G=0.06 61/143
PPARGC1A	Transcriptional coactivator, involved in energy metabolism	rs2290602	A>C	intron variant		Not known
PPP1R3B	Catalytic subunit of protein phosphatase-1 expressed in the liver; may be involved in glycogen synthesis regulation	Rs4240624	G>A	Intron variant		G=0.09 87/214
SAMM50	Component of the Sorting and Assembly Machinery in outer mitochondrial membrane	rs2143571	G>A	intron variant		A=0.32 19/701
SLC2A1	Glucose transporter	rs841856	G>T	Intron variant		T=0.19 05/415
		rs4658	C>G	3 prime UTR variant		G=0.36 18/788
SLC44A1	Fatty acid transporter	rs10820799	A>C	intron variant		C=0.12 40/270
SOD2	Mitochondrial protein, binds toxic superoxide products	rs4880	A>G	Missense	Val16Ala	G=0.37 05/806
SPINK1	Trypsin inhibiting enzyme, secreted by pancreatic acinar cells	rs17107315	T>C	Missense	N34S	C=0.00 60/13
SREBF1	Transcription factor, regulates LDL receptor and cholesterol biosynthesis genes	rs11868035	G>A	Splice region variant		A=0.46 92/102 2
SREBF2	Transcription factor, regulates cholesterol homeostasis	rs133291	C>T	intron variant		T=0.26 49/577
STAT3	Transcription activator, responds to cytokines and growth factors	rs9891119	A>C	Intron variant		C=0.37 14/809
		rs2293152	C>G	Intron variant		G=0.37 92/825
		rs6503695	T>C	Intron variant		C=0.33 15/722
TCF7L2	Transcription factor,	rs7903146	C>T	Intron variant		T=0.21

	role in glucose homeostasis, Wnt signalling pathway					81/475
TM6SF2	Transmembrane 6 superfamily member 2	rs58542926	C>T	Missense	Glu167Lys	T=0.06 67/334
TMPRSS6	Protease, involved in liver matrix remodelling	rs855791	A>G	Missense	Val736Ala, reduced enzymatic activity	A=0.39 81/867
TNF	Proinflammatory cytokine	rs361525 (*TNFA*)	G>A	Upstream gene variant		A=0.05 05/110
		rs1800629 (*TNF2*)	G>A	Upstream gene variant		A=0.09 55/208
		rs1799964	T>C	Upstream gene variant		C=0.20 02/436
		rs1800630	C>A	Upstream gene variant		A=0.14 51/315
		rs1799724	C>T	Upstream gene variant		T=0.09 73/211
UCP3	Mitochondrial anion carrier protein	rs1800849	G>A	utr variant 5 prime		A=0.20 20/439

MAF: Minor allele frequency; RAAS: renin-angiotesin-aldosterone-system

References

[1] Sanyal AJ, American Gatroenterological Association. AGA technical review on nonalcoholic fatty liver disease. Gastroenterology 2002;123:1705-1725.

[2] Matteoni CA, Younossi ZM, Gramlich T, Boparai N, Liu YC, McCullough AJ. Nonalcoholic fatty liver disease: a spectrum of clinical and pathological severity. Gastroenterology 1999;116:1413-1419.

[3] Teli MR, James OF, Burt AD, Bennett MK, Day CP. The natural history of nonalcoholic fatty liver: a follow-up study. Hepatology 1995;22:1714-1719.

[4] Brunt EM. Nonalcoholic steatohepatitis: definition and pathology. Semin Liver Dis 2001;21:3-16.

[5] Brunt EM, Janney CG, Di Bisceglie AM, Neuschwander-Tetri BA, Bacon BR. Nonalcoholic steatohepatitis: a proposal for grading and staging the histological lesions. Am J Gastroenterol 1999;94:2467-2474.

[6] Brunt EM, Kleiner DE, Wilson LA, Unalp A, Behling CE, Lavine JE, et al. Portal chronic inflammation in nonalcoholic fatty liver disease (NAFLD): a histologic marker of advanced NAFLD-Clinicopathologic correlations from the nonalcoholic steatohepatitis clinical research network. Hepatology 2009;49:809-820.

[7] Kleiner DE, Brunt EM, Van Natta M, Behling C, Contos MJ, Cummings OW, et al. Design and validation of a histological scoring system for nonalcoholic fatty liver disease. Hepatology 2005;41:1313-1321.

[8] Brunt EM, Kleiner DE, Wilson LA, Belt P, Neuschwander-Tetri BA, Network NCR. Nonalcoholic fatty liver disease (NAFLD) activity score and the histopathologic diagnosis in NAFLD: distinct clinicopathologic meanings. Hepatology 2011;53:810-820.

[9] Brunt EM. Pathology of nonalcoholic fatty liver disease. Nat Rev Gastroentero 2010;7:195-203.

[10] Brunt EM. Nonalcoholic steatohepatitis. Semin Liver Dis 2004;24:3-20.

[11] Neuschwander-Tetri BA, Caldwell SH. Nonalcoholic steatohepatitis; summary of an AASLD single topic conference. Hepatology 2003;37:1202-1219.

[12] Sheth S, Gordon F, Chopra S. Nonalcoholic steatohepatitis. Ann Intern Med 1997;126:137–145.

[13] Brunt EM. Histopathology of non-alcoholic fatty liver disease. Clin Liver Dis 2009;13:533-533.

[14] Lee R. Alcoholic and nonalcoholic steatohepatitis. In: Bloomer JR Z, Ishak K, editors. Clinical and pathological correlations in liver disease: Approaching the next millenium. Washington DC: American Association for the Study of Liver Disease; 1998. p. 2748–2783.

[15] Lee R. Non-alcoholic steatohepatitis: tightening the morphological screws on a hepatic rambler. Hepatology 1995;21:1742-1743.

[16] Bedossa P, Flip Pathology Consortium. Utility and appropriateness of the fatty liver inhibition of progression (FLIP) algorithm and steatosis, activity, and fibrosis (SAF) score in the evaluation of biopsies of nonalcoholic fatty liver disease. Hepatology 2014;60(2):565-75.

[17] HUGO Gene Nomenclature Committee (HGNC). HGNC Database. c2013 [cited 2014 01 June]; Available from: http://www.genenames.org

[18] GeneCards Human Gene Database. c2014 [cited 2014 01 June]; Available from: http://www.genecards.org

[19] National Center for Biotechnology Information. Gene. c2014 [cited 2014 01 June]; Available from: http://www.ncbi.nlm.nih.gov/gene/

[20] Flicek P, Amode MR, Barrell D, Beal K, Billis K, Brent S, et al. Ensembl 2014. Nucleic Acids Research 2014;42:D749-D755.
